# The Property, Preparation and Application of Topological Insulators: A Review

**DOI:** 10.3390/ma10070814

**Published:** 2017-07-17

**Authors:** Wenchao Tian, Wenbo Yu, Jing Shi, Yongkun Wang

**Affiliations:** School of Electro-Mechanical Engineering, Xidian University, Number 2 Taibai South Road, Xi’an 710071, China; wctian@xidian.edu.cn (W.T.); jshi@xidian.edu.cn (J.S.); ykwang@xidian.edu.cn (Y.W.)

**Keywords:** topological insulator, property, preparation, doping, application, photodetector, magnetic device, FET, laser

## Abstract

Topological insulator (TI), a promising quantum and semiconductor material, has gapless surface state and narrow bulk band gap. Firstly, the properties, classifications and compounds of TI are introduced. Secondly, the preparation and doping of TI are assessed. Some results are listed. (1) Although various preparation methods are used to improve the crystal quality of the TI, it cannot reach the industrialization. Fermi level regulation still faces challenges; (2) The carrier type and lattice of TI are affected by non-magnetic impurities. The most promising property is the superconductivity at low temperature; (3) Magnetic impurities can destroy the time-reversal symmetry of the TI surface, which opens the band gap on the TI surface resulting in some novel physical effects such as quantum anomalous Hall effect (QAHE). Thirdly, this paper summarizes various applications of TI including photodetector, magnetic device, field-effect transistor (FET), laser, and so on. Furthermore, many of their parameters are compared based on TI and some common materials. It is found that TI-based devices exhibit excellent performance, but some parameters such as signal to noise ratio (S/N) are still lower than other materials. Finally, its advantages, challenges and future prospects are discussed. Overall, this paper provides an opportunity to improve crystal quality, doping regulation and application of TI.

## 1. Introduction

With the development of science and technology, the exploration of the microscopic world has been deeply carried out, especially in the field of condensed matter physics and nanomaterials. One important research field in condensed matter physics is the phase of matter and the classification. Before the topological insulator (TI) is found, the phase of the substance is generally described by spontaneous breaking of symmetry. However, TI does not belong to this category [1,2]. That is to say, they should be described by a new topological order rather than symmetric breaking theory, long-range correlation and local order parameter [3]. TI is a general concern of science because its discovery is of great value to basic physics research and many semiconductor devices [4]. TI is another significant discovery after graphene, which will inevitably lead to the upsurge of research in the scientific community [5].

After the discovery of the Hall effect, the German physicist K.V. Klitzing won the 1985 Nobel Prize in Physics because the discovery of the quantum Hall effect (QH) 100 years later [6]. The QH is the Hall effect under the strong magnetic field (about 18 T) and low temperature (about 12 K). After that, physicists D.C. Tsui, R.B. Laughlin and H.L. Stormer applied a stronger magnetic field to the QH and found the fractional quantum Hall effect. The reward for this research was the 1998 Nobel Prize in Physics [7]. Since then, condensed matter physics has attracted a lot of attention in science. This also formed the basis for the discovery of TI [8].

Although the QH was found, it involves high cost as a result of strong magnetic field and low temperature environment. This limits its development. Therefore, the QH without specific environment was strongly required. Finally, the quantum spin Hall effect (QSH) was found in 2005, marking the beginning of TI exploration [9]. Through the experiment, it is found that the QSH can show an electronic state similar to the QH by the inherent nature of strong spin-orbit coupling effects rather than specific environment. The discovery of the QSH has raised new discussion and caused a research boom in science.

The study of TI did not cease over the period of more than decade. The TI has the QSH states at room temperature, which makes it have application and engineering value. However, the current research on this material still remains in a theoretical stage such as its surface states of scanning tunneling microscope (STM), Z2 topological Invariants and first-principles theoretical calculations. The study of its preparation, doping and properties are also promising research fields. However, Fermi level regulation still faces a challenge. The production cannot reach industrialization. Some parameters such as electron mobility also lower than theoretical value because the discovery of the TI is late. However, these studies aim to explore the excellent nature of TI and then use it in practical application and engineering. At present, the practical application of TI is still in an initial stage, and the research on it is limited. Scientists and researchers are making an effort to improve the performance of TI-based devices. 

In this paper, the latest development, main characteristics and classification of TIs are firstly introduced. The common TI compounds are also introduced and analyzed. Then its preparation methods and impurities effect (magnetic and non-magnetic) are summarized analyzed, which focuses on its superconductivity and ferromagnetism. After that, this paper summarizes and discusses the practical applications of TI. As they mainly revolve some optoelectronic and semiconductor devices, the TI-based photodetector, magnetic devices, FET and laser are mainly discussed. In this paper, the parameters and structures of the TI-based devices are analyzed and compared with the common semiconductor materials such as graphene. The results show that the TI-based devices exhibit excellent performance. In addition, the devices with heterojunction and tunnel structure have broader prospects. TI is a kind of material that has research and engineering value because it has the characteristics of low energy dissipation and efficiency. At the end of the article, the future prospects and challenges are analyzed and predicted according to the current research status of TI. Through the summary and analysis of this paper, we hope that more scientists can contribute to the development and research of TI.

## 2. Overview of TI

TI is a kind of special material. In general, solid materials are divided into three categories: conductor, insulator and semiconductor. Conductor is conductive because it has electron-holes on the bulk conduction band. The free electrons can move without crossing the band gap. Insulator cannot be conductive because of its wide band gap between the valence band and conduction band. The electron must absorb enough energy to cross the gap and migrate to conduction band. The band gap of the semiconductor is between the conductor and the insulator. However, the TI cannot be simply classified as any of the above categories.

TI has bulk electronic state with narrow band gap, which determines that there are no free carriers inside the bulk states. However, it also has topologically protected metallic surfaces. This gapless surface state has Dirac point that can pass through the band gap, which means the surface of the TI is conductive [10]. This special surface state is formed due to its internal strong spin-orbit coupling effects and the time-reversal symmetry. This means that the TI can reduce or avoid the scattering effects of non-magnetic impurities [11]. It should be noted that the number of Dirac points on different TI surfaces is not the same, resulting in differences among TIs.

### 2.1. Development of TI

The developing time of the TI is short because the discovery of the QSH is late (only just over a decade). One important thing is that the TI has improved from two to three dimensions in such a short period of time. It has also developed three generations of TI. The development speed of TI has been rapid in recent years.

#### 2.1.1. Two-Dimensional TI

The study began with the two-dimensional (2D) topological state. This state theoretically derived from the 2D materials such as graphene and the uniform gradient of the 2D semiconductor system. The root way to get TI is to make the material cause bulk band inversion. In 2007, B.A. Bernevig, T. Hughes and S.C. Zhang successfully validated this theory [12]. The QSH could be obtained by changing the thickness of the HgTe layer in CdTe/HgTe/CdTe semiconductor quantum well [13]. This was mainly because the spin-orbit coupling effect of CdTe was relatively weak. Increasing the thickness of HgTe was equivalent to enhance the spin-orbit coupling of the HgCdTe system. When the thickness increased to about 6.5 nm, there was a bulk band inversion so that the p orbital was pushed over the s orbital in this system. Up to now, only Hg_1−x_Cd_x_Te has been proved to be a 2D TI by experiment.

#### 2.1.2. Three-Dimensional TI

At present, the development speed of three-dimensional (3D) TI is very rapid. The third generation of TI has been studied, and its stability has also been improved. The first generation was Bi_1−x_Sb_x_ binary alloy (x = 0.07~0.22). It was found that its ratio is not stable and it is not a pure chemical phase. In addition, its surface state and Fermi level intersect five times which suggests it is not stable [14]. The surface structure of Bi_1−x_Sb_x_ is complex with a narrow gap. Hence Bi_1−x_Sb_x_ is not suitable for research and application. Scientists have got much further in optimized TIs. Then the second generation of TI appeared, including Bi_2_Se_3_, Bi_2_Te_3_ and Sb_2_Te_3_ [15]. They are all hexagonal structures with narrow band gap [16]. In 2009, S.C. Zhang predicted the existence of the second generation TI. After that, Xia used angle-resolved photoemission spectroscopy (ARPES) to observe the surface state of a single Dirac cone and calculated the surface state by first-principles. This gave experimental support to the prediction [17]. These TIs have narrow bulk band gap and simple structure so they are easy to be studied and prepared. They are currently the most widely used TI. In general, the surface state of the TI is one dimension lower than the bulk structure. The Dirac electrons move on its surface. For 2D TI, electrons move along the boundary of the material. Bi_2_Se_3_, Bi_2_Te_3_ and Sb_2_Te_3_ only have one Dirac point that can pass through the band gap so the surface state has the Dirac cone [18]. Spectroscopies that appear in pairs are separated to the different surfaces of the TI by topology. If the local state density is calculated on the open boundary by constructing the largest local Wannier function, a single Dirac cone can be seen on the surface state. In summary, the second generation of TI has many properties better than the prior TI. (1) The x is no longer a variable. Stoichiometric ratio is easy to control, which makes it easy to prepare pure chemical phase; (2) Dirac point is singular. This means they are strong TIs; (3) Bulk band gap is large, which meets the requirement of experiment and research. These properties mean that Bi_2_Se_3_, Bi_2_Te_3_ and Sb_2_Te_3_ have great research value.

The third generation of TI is called the topological crystalline insulators (TCIs). In 2013, Liu et al. prepared TCIs with the calculation of band gap structure and topological band analysis [19]. Similarly, its bulk had the band gap, but there was a boundary state with spin filter properties protected by mirror symmetry (001) on the edge. They had an even number of Dirac cones, which was protected by the mirror symmetry of the lattice instead of time-reversal symmetry. This novel topological phase could be achieved on the films of SnTe and Pb_x_Sn_1−x_Se(Te) (001). The third generation of TI had a more excellent feature, namely that their band gap could be controlled. By controlling the electric field that was perpendicular to the film, the mirror symmetry of the system could be destroyed, resulting in a controllable band gap on the edge state. This was different from the second-generation TI, which had multiple surface states. For example, SnTe (p-type) has four states on (100), (110) and (111) surfaces. SnTe is a rocksalt structure. Its specific surface orientation allows an even number of Dirac cones. In addition, its surface state is gapless, while the bulk has narrow band gap. TCIs could be used in many devices such as the photodetector, making TI a kind of promising material [20]. In 2017, Volobuev et al. from Johannes Kepler Universität found that by adjusting the doping amount of Bi atoms, the epitaxial Pb_1−x_Sn_x_Te (111) film could be induced to exhibit a large and controllable Rashba effect [21]. This effect was closely related to the direction of the distance vector. It should be noted that most systems could not do this. What is more, this method could improve the carrier mobility of TI (up to 10,000 cm^2^/V·s) and enhance the p-type character.

#### 2.1.3. New Generation of TI

With the development of TIs, some new topological compounds such as ZrTe_5_, HfTe_5_ and Weyl semimetals (WSMs) have aroused the interest of scientists. 

In 2014, Weng et al. predicted that single-layer ZrTe_5_ and HfTe_5_ were large-gap 2D TIs [22]. Their direct and indirect bulk band gaps were 0.4 eV and 0.1 eV respectively. Interestingly, the topological characteristics remained constant over a wide range of lattice strains (10% compression to 20% stretch), which could be applied to different substrates and applications. As for 3D ZrTe_5_ and HfTe_5_, their phase change points were between strong and weak TIs resulting in topological phase transition under a slight change of pressure or temperature [23]. After that, Yuan et al. observed and confirmed the existence of Dirac fermions in ZrTe_5_ [24]. In addition, the electronic structures of ZrTe_5_ and HfTe_5_ were similar to Dirac semimetal (DSM). In 2016, Li et al. observed the chiral magnetic effect and measured the magnetotransport in ZrTe_5_ [25]. The results showed that a negative magneto-resistance emerged when the magnetic field was parallel with the current because of the transmutation from DSM to WSM induced by current and magnetic field in a specific direction. After that, Qiu et al. studied the optical and electrical properties of few-layer ZrTe_5_ and demonstrated that a 50% of difference along different in-plane directions such as hole mobility (3000 and 1500 cm^2^/V·s along the a-axis and c-axis) [26]. Furthermore, superconductivity of ZrTe_5_ was induced by pressure (6.2 Gpa). In contrast, this material had two superconducting phases and the second phase was induced by pressure above 21.2 Gpa. The transition temperature (T_c_) peaked at 4.0 K at 14.6 Gpa [27]. This material could also be used to study quantum oscillation because of the strong electron interaction [28]. It should be noted that the property of HfTe_5_ was similar to ZrTe_5_. It also had superconductivity induced by pressure (from 5.5 Gpa to 35 Gpa). The highest T_c_ was about 5 K under 20 Gpa [29]. Moreover, chiral anomaly and ultrahigh mobility (1.82 ± 0.01 × 10^6^ cm^2^/V·s) were observed in HfTe_5_ crystals [30]. ZrTe_5_ and HfTe_5_ are transition metal compounds so the different metal-doped materials are easy to obtain, which provides an opportunity to achieve physical regulation. 

In addition to ZrTe_5_ and HfTe_5_, WSMs are also a class of advanced and promising materials. This kind of topological semimetal has many excellent properties. It has discontinuous and topologically protected surfaces Fermi arcs as well as Weyl nodes in the bulk [31]. This novel state can exist without bulk gap and it is also affected by the positions of Weyl points. The WSM phase is between the normal and topological phase [32]. What is more, the efficient band is a linear Dirac-type dispersion with low energy. The exciting property is the chiral anomaly and a series of electromagnetic responses induced by chiral anomaly. In 2015, Weng et al. used first-principle calculations to find a family of non-magnetic WSMs (TaAs, TaP, NbAs and NbP) [33]. It is worth mentioning that these non-magnetic topological semimetals could be used to observe chiral anomaly and Fermi arc, which was impossible in magnetic WSMs such as Y_2_Ir_2_O_7_ and HgCr_2_Se_4_ because of the existence of magnetic domain. S. Xu reported that the Fermi arcs, Weyl fermion cones and Weyl nodes could be directly observed by photoemission spectroscopy in TaAs bulk [34]. After that, the negative magnetoresistance induced by chiral anomaly and time-reversal invariant of WSM was proved by Huang and Zhang et al. [35,36]. Like ZrTe_5_ and HfTe_5_, WSM such as NbP held large magnetoresistance (850,000% at 1.85 K and 250% at room temperature respectively) and high mobility (5 × 10^6^ cm^2^/V·s) [37]. It should be noted that the compounds mentioned are all type-I WSMs. The type-II WSMs such as WTe_2_ and MoTe_2_ that have tilted Dirac cone also exhibit novel characteristic [38]. When the magnetic field is applied in some specific directions, the material shows an insulator behavior, while it exhibits a conductor behavior in the other directions. It is also different from type-I WSMs that the magnetoresistance of type-II WSMs is related to the crystal orientation. This means that the resistivity will increase like a normal metal when the magnetic field and current flow along some specific crystal directions. However, it will decrease like WSMs in other orientations. In addition, the negative longitudinal magnetoresistance can be seen to result from the chiral anomaly. In 2016, Wang et al. confirmed this and suggested that WTe_2_ showed strong planar orientation dependence [39]. They also demonstrated that the ideal thickness of WTe_2_ sample was 7–15 nm, which was suitable to study the chiral transport characteristics. The Fermi arcs and phase of type-II WSM could be observed by ARPES in experiment [40,41,42]. There are many ways to study WSM. It can be seen as the extension of the 3D graphene, which can be studied by means of the theory and knowledge of graphene. Furthermore, it can be studied from the magnetically doped TIs model with layered stacking superlattice. Also, the research can begin from the Dirac equation. The Dirac fermion is split into a pair of Weyl fermions. Then further discussion has arisen regarding whether it is time-reversal symmetry breaking or spatial inversion symmetry breaking. Apart from the DSMs and WSMs, another semimetals called topological Node-Line semimetals (Cu_3_PdN and Mackay-Terrones crystals) also show an interesting property such that the band intersection forms a continuous curve in the momentum space [43,44].

### 2.2. The Main Properties and Characteristics of TI

TIs are a general concern of the scientific community because of their excellent properties and characteristics. This section describes the main features and properties of TI.

#### 2.2.1. Photon-Like Electron

In an ordinary conductor, the dispersion relation is non-linear. In contrast, TI is characterized by linear dispersion relation between the energy and momentum, which is like the propagating of photon. This property can enhance the performance of semiconductor devices resulting from thehigh sensitivity to the external electric field of the topological surface. Carrier Mobility is the generated average drift velocity under the unit electric field strength, which represents the carrier conductivity. The ideal TI has high mobility. Theoretically, the modulation doping technique could increase the mobility by an order of magnitude. So the TI has great potential in this area [45,46]. In practical application, high mobility makes a TI-based device have fast running speed and high cut-off frequency.

#### 2.2.2. Low Power Dissipation

In addition to high mobility, TI has another excellent property—low power dissipation. The resistance of the insulator is due to the presence of band gaps, while the resistance of the metal is due to the collision of electrons with phonons, impurities, and so on. The TI has a Dirac electron on the surface that can bypass the impurity and move in the original direction while encountering the impurities. Due to the spin of the electrons, the surface state of the TI has four degrees of freedom, which is twice as much as the 1D system without spin. When it encounters the impurity, the electrons move along the clockwise and counterclockwise direction, and the spin direction also reverses. The two scattering waves are coherently canceled, which is the principle of avoiding impurities. This nature greatly reduces the resistance. Meanwhile, the internal insulator prevents leakage of electricity. Therefore, TI-based devices can run at low power. Besides, the heating problem of the integrated circuit is serious with the increase of integration. If the TI is used to transmit information, it will be possible to fundamentally solve the heat problem.

#### 2.2.3. Spin-Polarized Electrons

The electrons of TI surface state have a spin-polarized structure. The spin is divided into two directions (up and down), which also increases the degree of freedom. This makes it possible to control the spin-polarized electrons in different directions. The spin-polarized electrons of the Dirac cone on the TI surface state can be observed by ARPES [47]. Although the TI has insulating bulk state, the surface has spin-dependent electrons with spin-momentum locking. Hence, this also contributes to the study of spin-electronic and magnetic devices.

#### 2.2.4. QSH

The QSH is the most representative property of TI. The surface state of the TI can be used to carry out the experiments of QSH, and it can also be applied to study the half-integer quantum Hall effect. Furthermore, the fractional charge and magnetic monopolar can even be studied [48,49]. The decoherence will happen while observing the ordinary quantum states because the normal quantum state is unstable. The wave function probability amplitude immediately turns from successive to discrete distribution after slight perturbations. However, the quantum state of the TI is very stable and will not be affected by slight perturbations, which makes it possible to utilize TI for quantum computation.

### 2.3. Classification of TI

There are three ways to classify the TI. The first one is classified according to the dimensions of TIs. The second one is sorted by the parity of Dirac points. The latter determines whether the TI is strong or not. The last one is about the symmetry.

#### 2.3.1. Classification by Dimensions

TIs can be divided into 2D and 3D structures. The ZrTe_5_ and HfTe_5_ have a single layer with a large gap in the bulk. HgCdTe quantum well structure is a typical 2D TI. However, it tends to be suitable for experimental research of the principle so its application value is limited. In contrast, the 3D TIs are more promising because the stoichiometry is relatively stable and the chemical phase is pure. This means that the compounds are easy to be prepared. In addition, the size of its band gap is more moderate (0.35 eV), which makes it available to be studied. The 3D WSMs also have novel quantum and physical properties, which are capable of being used to study chiral anomaly, Fermi arc and other theories in condensed matter physics. For 2D TI, the electronic state is the one-dimensional edge, and for the 3D TI, its topological state is 2D. That is to say, its exterior surface is the topological metallic state, while its bulk is the ordinary insulator. The reason why the dimension of surface state is lower than the bulk dimension is that the topological state acts as the “transition state” between bulk and surface.

#### 2.3.2. Classification by Parity of Dirac Points

**Figure 1:**
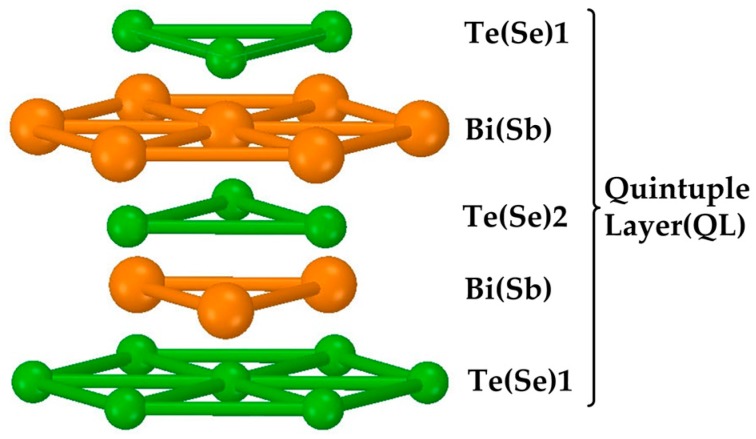
The quintuple layer of topological insulators (TIs).

TI can be divided into two categories: weak TI and strong TI. For the weak TI such as Sb_2_Se_3_, the surface Brillouin zone has an even number of Dirac points. Strong disorder makes the electron localized on the surface [50]. When it comes to strong TI, the surface is a system that has an odd number of Dirac points. Electron localization is not affected by non-magnetic disorder on the surface, resulting in the perfect metallic surface state. This also explains why although almost all compounds have spin-orbit coupling, only a little part of them are the TI. Generally speaking, the TI that is always mentioned refers to the strong one including Bi_2_Se_3_, Sb_2_T_3_ and Bi_2_Te_3_. They have similar molecular structure so we only take Bi_2_Se_3_ as example. Figure 1 shows the quintuple layer (QL) of TIs. There are same atomic species on the same layer. Each quintuple layer consists of five atoms with Se (1)-Bi-Se (2)-Bi-Se (1). Se (1)-Bi is bonded with covalent bonding and ionic bonding. Bi-Se (2) is bonded with covalent bonding, while the Se (1)-Se (1) is combined with van der Waals [51]. So Bi_2_Se_3_ is prone to dissociate between Se (1) atom layers. Their space groups are all R(-3)M. Their common JCPDS Card Numbers are 33-0214, 15-0874 and 15-0863 respectively.

#### 2.3.3. Classification by Symmetry

TIs such as Bi_2_Te_3_, Bi_2_Se_3_ and Sb_2_Te_3_ have the time-reversal symmetry. The gapless topological surface has Dirac electrons. TCIs hold boundary state with spin filter properties protected by mirror symmetry on the edge. The crystal surface has an even number of Dirac cones. However, WSMs have the time-reversal symmetry and low crystal symmetry, which holds Weyl fermions in the bulk as emergent quasiparticles and Fermi arcs on the surface. The type-I WSMs respect Lorentz symmetry, while the type-II WSMs do not. In addition, the Weyl points of the type-II WSMs appear at the boundary of the electron and hole pockets.

### 2.4. Compounds of TI

Although there are 2D, 3D, strong and weak TIs, the compounds that are usually used for study and application are less. Bi_2_Se_3_, Sb_2_Te_3_ and Bi_2_Te_3_ are the most popular strong TIs. Table 1 shows the properties and parameters of Bi_2_Se_3_, Sb_2_Te_3_ and Bi_2_Te_3_, and it is obvious to see the differences among them. It should be noted that the figure in the Table 1 is not the ideal parameter of TI.

Some results can be seen from Table 1. (1) The physical properties of the three TIs are very similar. The reason for this is that they are composed of elements of the V and VI main groups, and their lattice structures are also consistent. Furthermore, the atoms are mainly bonded with covalent bonding, but also mixed with ionic bonding. Each of the five atoms between the QLs is like graphite, which is connected with van der Waals; (2) The band gap of Bi_2_Se_3_ is the largest among these three commonly used TIs so it is better prepared and studied; (3) Bi_2_Te_3_ has the highest electron mobility and hole mobility, indicating that it is least affected by impurity. Hence, it is suitable for devices that require high mobility; (4) Similarly, the thermal conductivity of Bi_2_Te_3_ is also the highest, reaching 3.00 W/m·K, suggesting that its thermal property is the best. However, the thermal conductivity of Sb_2_Te_3_ is the lowest, which is only half as high as Bi_2_Te_3_; (5) Their surfaces have only one Dirac point, which also reduces the difficulty of research.

## 3. Preparation and Doping of TI

TIs have a variety of preparation methods, but the advantages and disadvantages of each method are different. In practical applications, the low-cost preparation method that can prepare high purity of TI is always selected. Furthermore, doping has a significant effect on the performance of TIs. Impurities are generally unavoidable while preparing. These impurities have different effects on the performance of the TIs. In addition, impurities can also be artificially controlled to change the parameters of the TIs resulting in desired properties. For instance, both n-type and p-type 3D TIs can be obtained by controlling the doping element and amount. This chapter introduces the mainstream TI preparation methods and discusses the influence of doping on them.

### 3.1. Preparation Methods of TI

TIs are mainly prepared by mechanical exfoliation, molecular-beam epitaxy (MBE), chemical vapor deposition (CVD), solvothermal synthesis and metal-organic chemical vapor deposition (MOCVD).

#### 3.1.1. Mechanical Exfoliation

The molecular structure of 3D TI is special—each two Bi atoms and three Se atoms constitute the basic QL. Each of the two QLs is connected by van der Waals, which is a weak intermolecular force. The crystal is easy to dissociate between layers under the external force. The graphene can be prepared by stripping the graphite layer-by-layer. Similarly, TI layers can be obtained by exfoliating the TI. 

**Figure 2:**
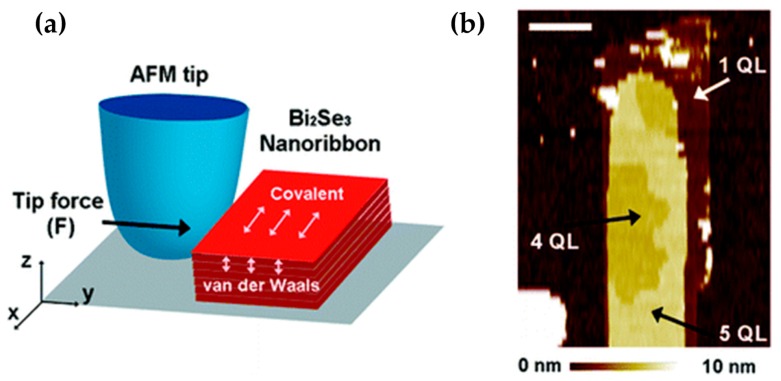
(**a**) Schematic of atomic force microscopy (AFM) exfoliation of layered structure nanomaterial-Bi_2_Se_3_ nanoribbon; (**b**) Topographic image of an exfoliated nanoribbon with three different thicknesses (1 QL, 4 QLs, 5 QLs) in different regions [62].

In 2010, Hong et al. from Stanford University successfully produced ultra-thin Bi_2_Se_3_ films [62]. They synthesized the Bi_2_Se_3_ nanoribbon using vapor-liquid-solid (VLS) method. Ultra-thin Bi_2_Se_3_ nanoribbons with the thickness of several QLs (minimum 1 QL) were completely stripped out from the thick nanoribbons (over 50 QLs) thorough the tip of atomic force microscopy (AFM). This method was judged as a controllable mechanical exfoliation. Figure 2a is the schematic diagram of this method. The horizontal tip force (y direction) was applied to the side of the nanoribbon to destroy the in-plane covalent bonding. Figure 2b shows the morphology of the three kinds of thickness (1 QL, 4 QLs and 5 QLs) in different regions of the stripped nanoribbons. In 2010, V. Goyal found that the thermoelectric property of TI thin films was improved and stacked by mechanical exfoliation [63].

#### 3.1.2. Molecular-Beam Epitaxy

MBE can be used to prepare semiconductor thin films and photoelectric thin films. It is an important part of modern epitaxial growth technology. In ultra-high vacuum conditions, one or several components of the thermal atoms or molecular beams are injected to the surface of the heated substrate. Then they are reacted with the surface of substrate, resulting in the deposition of single crystal film. After the molecular beam has exchanged energy with the substrate, it will experience the surface migration, adsorption, and nucleation. Finally, it will grow into film. This process involves both physical and chemical changes. The compound is combined with the substrate in the formation process.

In 2011, Krumrain and Mussler et al. from Germany studied the growth of the TI Bi_2_Te_3_ on Si (111) substrate by MBE [64]. They found that the diffusion and desorption of the adsorbed Te atoms could be compensated under the Te overpressure condition. This could achieve the layer-by-layer growth of Bi_2_Te_3_. In addition, proper Te flux must be kept during the cooling period after deposition to prepare the monolayer Bi_2_Te_3_ in good quality. Furthermore, more than 75% of the surface layer could be covered under the optimized growth condition. AFM scan data showed the thickness of only 1 nm (about 1 QL) [65]. In the same year, Wang et al. found that Bi_2_Te_3_ film (undoped) with different carrier types could be fabricated at different growth temperatures by MBE method. It showed n-type under 590 K temperature whereas p-type under 630 K [66]. By controlling the growth rate, the defects could be reduced. In addition, the growth rate was only affected by the Bi (Sb) flux. The change of growth parameters could also lead to different carrier types. It should be noted that optimized growth conditions could enhance the mobility of the material.

**Figure 3:**
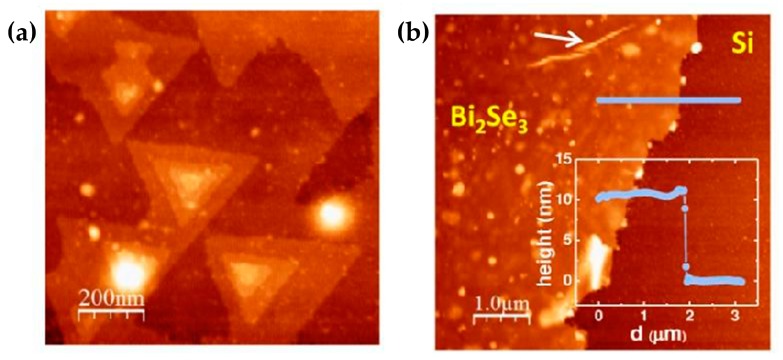
Characterization of the transferred film: (**a**) AFM image of the 10 QL film transferred to Si (111), exhibiting the pristine morphology of the transferred film; (**b**) A large area AFM image (5 × 5 μm^2^) showing the edge of the transferred film; the inset shows the height profile of the film along the line drawn on the image [67].

In the year of 2014, Bansal et al. transferred the entire Bi_2_Se_3_ film to any substrate and maintained its original morphology and crystallinity [67]. Besides, the transferred film had lower carrier concentration than before. Figure 3 shows the characterization of the transferred film. By observing the images with AFM, it can be seen that the transferred film still maintained the high integrity. Figure 3a confirms that the chemicals used in this process did not affect the quality of the film. Figure 3b shows that the measured height (~10 nm) is consistent with the original thickness of 10 QL. The pointed portion of the arrow might be the micro wrinkles in the edge of the transferred film.

#### 3.1.3. Chemical Vapor Deposition

CVD is a common method for preparing high quality TIs. Linear, ribbon and sheet-like nanomaterials can be prepared by this method. The reactant evaporates because of the high temperature. Then the generated material deposits on the surface of the heated substrate through the gas transmission. This method can fabricate a large area of the material. Physical vapor deposition (PVD) only has phase change. In contrast, CVD is different because it also accompanies chemical changes. The main mechanism of this is the gas-liquid-solid phase method.

In 2014, Liu et al. fabricated high-quality multilayer Bi_2_Se_3_ film with asymmetric, elongated hexagonal structure using CVD [68]. In the experiment, high purity of Se and Bi powders were used under the protection of Se-rich gas. Results showed that Au particles were only used as nucleation at the initial stage of Bi_2_Se_3_ nanostructures at low temperatures. The Se powder in the source material helped to maintain the stoichiometric ratio and reduced the Se vacancies. Also, the Bi_2_Se_3_ nanoribbons grew along the lateral direction. 

In 2016, Sun et al. investigated the effect of the seed layer on the graphene-Bi_2_Se_3_ nanoplates mixed with Dirac material when using the CVD method [69]. The results of SEM, Raman and X-ray diffraction (XRD) showed that Se seed layer provided good nucleation sites. Furthermore, they found that sufficient Se atoms could fill the Se vacancies just like the conclusion of M. Liu’s study in 2016. Se plate, as the role of isolation layer, could also prevent the reaction between graphene and Bi_2_Se_3_. The existence of Se seed layer significantly improved the quality of graphene-Bi_2_Se_3_ nanoplates. Wang et al. used CVD to prepare ultrathin Pb_1−x_Sn_x_Te nanoplates (40 nm) on SiO_2_/Si substrate in the same year [70].

In 2017, Lee et al. successfully grew Bi_2_Te_3_ and Sb_2_Te_3_ films on graphene substrate [71]. By observing the microstructure of the deposited material, an interesting fact was determined—the surface of the material was smoother than conventional SiO_2_ or Si substrates when the growth temperature was 250 to 300 °C. In addition, the film grew mainly along the c-axis. However, in other temperature ranges, the crystal quality was difficult to guarantee. Therefore, the need for strict temperature control was slightly worse than the traditional substrate.

#### 3.1.4. Solvothermal Synthesis

Solvothermal synthesis is a new generation method to fabricate nanomaterials developed by hydrothermal method. Under high temperature and pressure condition, the material in the solution grows into nucleation in the presence of the catalyst in the sealed container. Finally, it crystallizes into nanomaterials. Solvent used in solvothermal synthesis is not water, but organic or non-aqueous solvents such as organic amines and alcohols.

**Figure 4:**
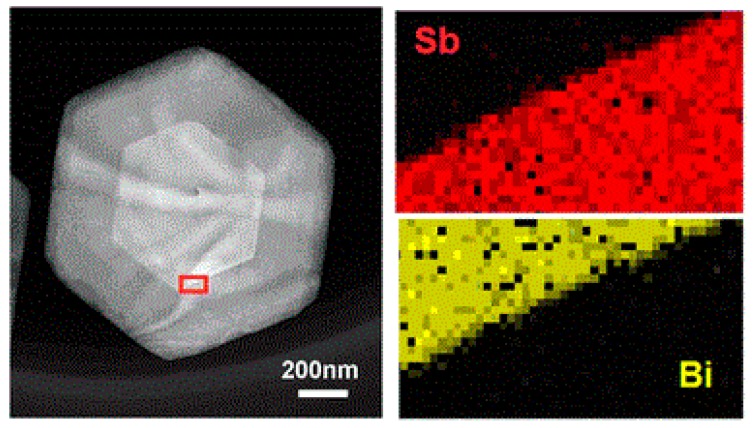
TEM image of lateral heterojunction and junction of Bi and Sb [72].

In 2015, Fei et al. used the two-step solvothermal synthesis to prepare the horizontal heterojunctions of Sb_2_Te_3_/Bi_2_Te_3_ [72]. The two crystal-matching interfaces were well separated. TEM images also showed the heterojunction growing along the horizontal direction. Figure 4 shows the interface of the heterojunction of the material. It can be seen that Sb and Bi layers formed a clear boundary, indicating that the material had high crystal quality.

Solvothermal synthesis cannot only prepare TI heterojunctions, but also achieve doping during the preparation process. In 2016, Mao et al. from Soochow University synthesized layered S-doped Bi_2_Se_3_ microspheres using this method. These microspheres were assembled from stacked nanosheets [73]. The microstructure of layered S-doped Bi_2_Se_3_ was synthesized using BiCl_3_ and selenium powder in the presence of mercaptoethanol. The doped material could be used as the anode of a lithium-ion battery. After 100 cycles, there was still a specific capacity of 109.4 mAh/g. The figure was about twice as high as the TI or its composite material [74,75,76].

#### 3.1.5. Metal-Organic Chemical Vapor Deposition

MOCVD is a new technology developed by vapor phase epitaxy (VPE). The source materials for crystal growth are organic compounds (group III, group II elements) and hydrides (group V, group VI elements). The gas phase epitaxy is carried out on the substrate by thermal decomposition reaction, thereby growing compound semiconductors and single crystal film. MOCVD is a superfine processing technique for the preparation of semiconductors and thin film materials.

**Figure 5:**
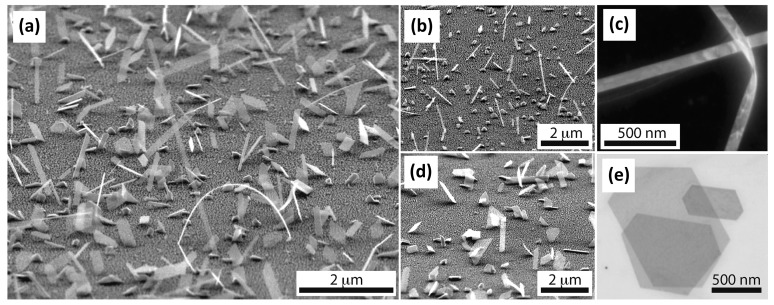
Bi_2_Se_3_ nanoribbons and platelets: (**a**,**b**,**d**) SEM images of as-grown samples and (**c**,**e**) images obtained after deposition on TEM grids [78].

In 2012, Alegria et al. from Princeton University used MOCVD to prepare ultra-thin Bi_2_Se_3_ single crystal nanoribbon as long as 10 μm [77]. This length could be up to three to four times as much as that of the solvothermal synthesis. MOCVD can also fabricate large-area TI like CVD. They optimized MOCVD by changing the Se/Bi partial pressure ratio (r = 33), the growth time (t = 15min) and the substrate temperature (T = 470 °C). This reduced the effect of Se vacancy on bulk conduction. Figure 5 is the SEM image of Bi_2_Se_3_. It can be seen from the figure that the crystal quality of TI was better than that in other conditions after optimizing the growth parameters. Figure 5a shows the growth on the Si (100) substrate with 5 nm Au seed layer for 15 minutes (T_g_ = 470 °C, P = 100 Torr, 600 sccm H_2_ carrier gas, precursor ratio r = 33). Figure 5b shows the nanoribbon (precursor ratio r = 12) resulting in a narrow band with a defined width of 70 ± 20 nm. Figure 5c shows a dark field scanning transmission electron microscopy (STEM) image of two nanoribbons with 85 × 10 nm^2^ cross section. Figure 5d shows that the product at r = 30 and growth temperature of 490 °C. Figure 5e is the ~1 μm^2^ crystal product observed by STEM. In the same year, Alegria et al. conducted a study of the structure and electrical properties of the prepared crystals [78]. The results showed that the resistivity reached 4 mΩ·cm, the phase coherence length was 178 nm and the highest spin-orbit length stood at 93 nm. 

In 2012, Cao et al. used MOCVD to prepare the wafer-scale Bi_2_Te_3_ thin film on the GaAs (001) substrate and measured its electrical properties [79]. The results showed that the bulk carrier mobility was 350 cm^2^/V·s at 30 K. Then it increased over twentyfold (7400 cm^2^/V·s) at 15 K. It can be known that in addition to the growth time, the temperature and substrate also had impact on the MOCVD. In 2014, Bendt et al. used Et_2_Te_2_ and i-Pr_3_Sb for growing smooth and high-quality Sb_2_Te_3_ films on a c-oriented Al_2_O_3_ (0001) substrate [80]. However, the preparation temperature was 500 °C which was slightly higher than that in L.D. Alegria’s experiment.

#### 3.1.6. Other Synthesis Methods

In addition to the above preparation methods, there are many other syntheses. In 2014, Shen et al. used VLS and vapor-solid growth methods to grow SnTe nanoplates that the crystallographic surfaces (100 and 111) could be selected [81]. The structural phase transition was also observed from rock salt at high temperature to rhombohedral structure at low temperature. However, the thickness of SnTe reached a hundred nanometers, which limited its observation of topological surface. In 2016, Lee et al. demonstrated that high-quality Bi_2_Te_3_ thin film could be fabricated by PVD on Al_2_O_3_ substrate by a temperature-gradient between two controllable heaters inside a furnace [82]. This method was relatively cost-efficient and the parameters of TIs were acceptable such as the electron mobility (192.4 cm^2^/V·s at 3 K). The pulsed laser deposition (PLD) is another common method used in experiments. The substrate temperatures of PLD were optimized and the best temperature was about 230 °C for Bi_2_Se_3_ thin films on SrTiO_3_ (STO) substrate [83].

#### 3.1.7. Comparison of Different Preparation Methods

Section 3.1 describes several major methods for preparing TIs. Table 2 is the comparison of these methods. It shows the advantages and disadvantages of different preparation methods, which can help to select the most suitable preparation method for the research. It can be seen from Table 2 that the different structures of product were fabricated by CVD under the low temperature (LT) and high temperature (HT) [68,69]. The CVD is regarded as a promising method because it can fabricate large-scale TIs. MBE is a common preparation method used in the laboratory. Furthermore, solvothermal synthesis is utilized in research of the doping problem. Currently, MOCVD has a promising future. It can be used to prepare high-quality TI, but the cost is high. The selection standards of the research are generally the precision, purity, crystal quality and structure. However, the selection criteria of industry are generally based on cost, safety, repeatability, which is beneficial to produce large-scale product.

### 3.2. Effect of Doping on TI

Doping is very important for many materials including TI. For example, the thermoelectricity coefficient and surface electron mobility of Bi_2_Se_3_ could be greatly improved (up to two orders of magnitude) by appropriate doping [91]. Doping impurities are mainly divided into non-magnetic (Ag, Ga, etc.) and magnetic elements (Fe, Mn, etc.). Because of the time-reversal symmetry, the non-magnetic impurities have little influence on TI. In contrast, the magnetic elements can open the band gap of surface state and destroy the protection of time-reversal symmetry. Therefore, some physical effects can occur such as magnetic monopole and QAHE [49,92]. Furthermore, doping is generally carried out during the preparation of the material. In addition to artificial doping, the intrinsic defect of the material is always unavoidable such as Se vacancies. Increasing the proportion of Se atoms or adjusting the annealing process could decrease the Se vacancies concentration. Also, it is possible to reduce the effect of heavy electron doping which results from the Se vacancies. The Se vacancies concentration was suppressed by Sb^3+^. The same effect could be achieved by introducing atoms with small ion radius such as S^2−^, O^2−^ [93]. Se vacancy, also known as lattice vacancy, is a typical point defect of Bi_2_Se_3_. It is the normal array that an atom is vacant (Schottky defect). What is more, there is a kind of atom called the gap atom that occupies the lattice gap position. It should be noted that the surface electron concentration of Bi_2_Se_3_ doubled when it was exposed to the air for a long time. The Dirac Hall doping also turned into electron doping. The reason for this might be that the TI tended to be oxidized into BiO_x_ compound in the air. Thereby this reduced the surface quality of TI [94]. Furthermore, the charge carrier types and concentration of the TI could be controlled by doping [95]. It can be known that the impurities influence TI's band gap and Dirac point. Overall, it is still a challenge that how to maintain the surface state of TI while doping. 

#### 3.2.1. Non-Magnetic Impurities

Non-magnetic impurity is a common type of doping. Its main elements are Ag, Ga, Mg, In, Cu, Ca and Sb. In general, different impurities have various effects on TIs. For example, they influence the carrier concentration, magnetoresistance and thermoelectric coefficient of materials. Also, superconductivity can be obtained by non-magnetic impurity doping. Several impurities are discussed below.

(1) Ag: Ag atoms have two ways of doping [96]. The first one is called inserted doping. This means that Ag atoms can be doped into the layers of TI resulting in the increase of lattice constant. For instance, if Ag atoms are inserted between QL-QL of the Bi_2_Se_3_, it is called the Ag_x_Bi_2_Se_3_. This may be due to the atomic radius of Ag (1.44 Å) being smaller than that of Bi (1.70 Å). Another way is called substituted doping. The Ag atoms replace the original atoms in the TI resulting in the decrease of lattice constant. In the case of Bi_2_Se_3_, the compound after doping in this way is expressed as Ag_x_Bi_2−x_Se_3_. The lattice compatibility of Ag_x_A_2_B_3_ (A_2_B_3_ represents Bi_2_Se_3_, Bi_2_Te_3_ or Sb_2_Te_3_) is higher than that of Ag_x_A_2−x_B_3_. However, the two doping methods have the same crystal structure. Meanwhile, they are generally coexistent, but their proportions are different.

The preparation method of Ag-doping used for research is generally the solvothermal synthesis. In order to improve the single crystal quality and cleavage, doping should meet the condition—Ag_x_A_2−x_B_3_ (x < 0.12) and Ag_x_A_2_B_3_ (x < 0.15) [97]. When the Ag amount was saturated, the excess Ag atoms would react with the remaining Se atoms to form the Ag-Se compound. Furthermore, some parameters of the Ag-doped TI would be improved such as the thermoelectricity coefficient, conductivity, thermal conductivity and resistivity. For example, Ag_0.1_Bi_2_Se_3_ had a conductivity of up to 3500 cm^2^/V·s (T = 50 K) [97].

**Figure 6:**
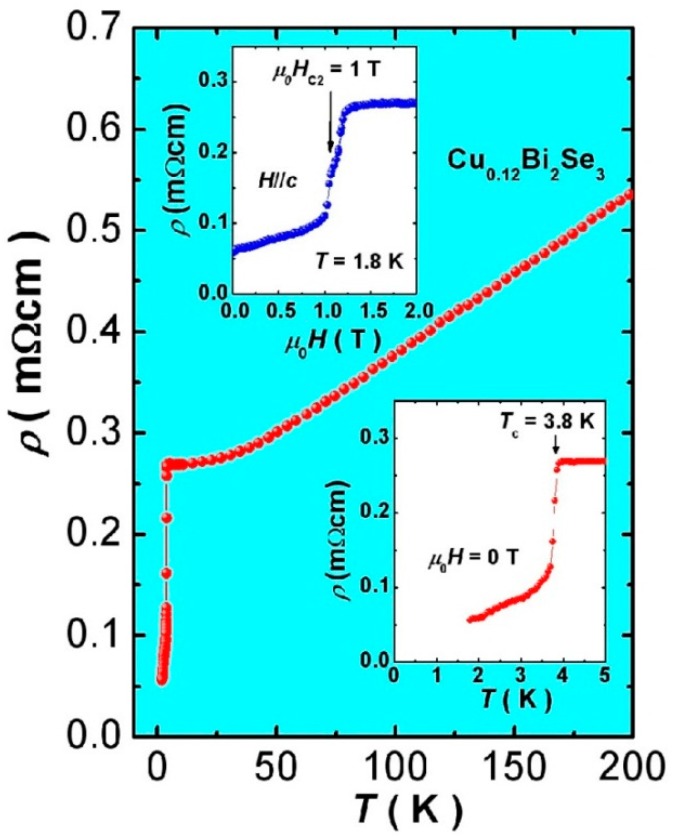
The temperature dependent resistivity of a Cu_0.12_Bi_2_Se_3_ single crystal [72].

**Figure 7:**
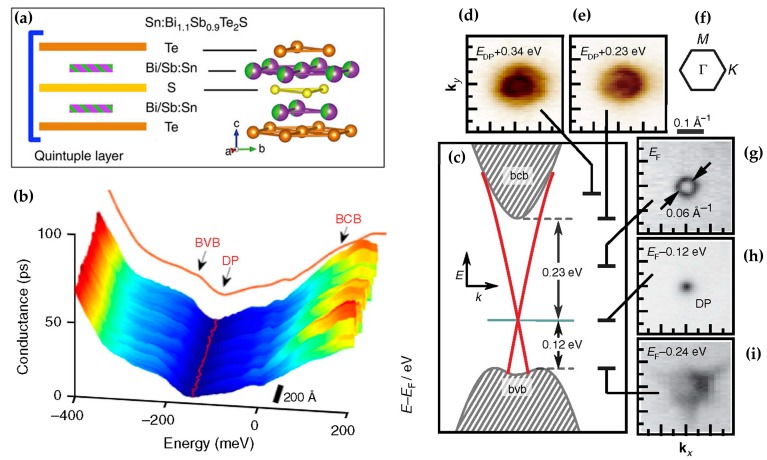
(**a**) The schematic of a QL for Sn:Bi_1.1_Sb_0.9_Te_2_S and a QL obtained by structure refinement of a powdered specimen; (**b**) The differential conductance (dI/dV) along a line shows only small fluctuations in the electronic surface structure (V_bias_ = −600 mV and I = 60 pA); (**c**) A schematic diagram for the electronic structure of Sn-BSTS in the vicinity of the Fermi energy derived from the angle-resolved photoemission spectroscopy (ARPES) data; (**d**,**e**) The DP E_DP_ for the K-deposition-generated electron-doped sample; (**f**) A schematic diagram for Brillouin zone with high symmetry points; (**g**–**i**) The constant-energy ARPES maps [106].

(2) Cu: Cu-doped TI has two chemical formulas that have same crystal type. The TI system still has metal conductive behavior when Cu atoms replaced Bi atoms. However, when the Cu atoms were inserted between QL-QL, the system exhibited superconductivity at low temperature (T < 3.8K) [98]. Similarly, the excess Cu atoms reacted with the remaining Se atoms to form Cu-Se compounds. The insertion of Cu atoms could alter the electrical transport properties of TIs. In 2010, Hor et al. successfully produced Cu_x_Bi_2_Se_3_ (x = 0.12~0.15). It showed superconductivity (electron concentration = 2 × 10^20^ cm^−3^) at 3.8K of T_c_ [99]. In the same year, Wray et al. studied the Cooper pairing theory of superconductivity [100]. Figure 6 shows the temperature dependent resistivity of the Cu_0.12_Bi_2_Se_3_ single crystal. It indicates that the T_c_ of superconductivity stood at 3.8 K. When the current was applied to the surface of TI, the resistivity of the Cu_0.12_Bi_2_Se_3_ changed. The above illustration is the magnetoresistance diagram at T = 1.8 K. Not only could Cu atoms make the TI appear to have superconductivity, but also Pd_x_Bi_2_Te_3_ reacted by Bi_2_Te_3_ and Pd showed superconductivity at T = 5.5 K [101].

(3) Ga: Ga sample is not easy to weigh and store because its melting point is just 29.8 °C. Generally, the Ga_x_A_2−x_B_3_ can be prepared by properly mixing A_2_B_3_ and Ga_2_Se_3_. Ga doping did not change TIs’ lattice structure, but it reduced its lattice parameters. This is mainly because the atomic radius of Ga (1.40 Å) is shorter than the atomic radius of Bi (1.70 Å) [102]. Se vacancy made TI have the n-type carrier type. Furthermore, the carrier concentration and carrier type of Ga-doped TI did not change. With the increase of x, the electron mobility of Ga_x_A_2−x_B_3_ rose first and then dropped. The electron mobility reached the peak at 2350 cm^2^/V·s (T = 50 K) when x remained at 0.03 [97]. The reason for this might be too many Ga atoms, which destroyed the scattering mechanism. This would also increase the lattice defects and resistivity of TI.

(4) Mg and In: Impurities of Mg and In do not change the lattice structure of the common 3D TI so they are discussed together. Mg and In doping did not change the morphology and electrical transport properties of the common 3D TI. Therefore, they had little effect on the resistivity. However, In doping influenced the structure and transmission properties of the TCIs [103,104]. For example, different x had various effects on In_x_(Pb_1−y_Sn_y_)_1−x_Te [104]. When the doping amount reached 6%, it had the bulk insulation conductivity. However, it showed superconductivity as the concentration went to 10%. The transition temperature T_c_ was 3~5 K, which was close to that of Cu-doped TIs [99,103]. In recent years, there have been many articles about the superconductivity of In-doped TI. Although the doping method is similar, the result is slightly different due to the unique equipment and the preparation methods. For instance, the transition temperature was different with the same doping amount. The figure is compared in Table 3.

(5) Sn: In 2009, Chen et al. from Stanford University found that Sn was a good dopant for TIs [105]. They suggested that 0.67% Sn-doped Bi_2_Te_3_ was the 3D TI with a single Dirac cone and a large bulk band gap (0.325 eV). With the study of doping research, some scientists have found that the new TI can even be fabricated by the use of doping—Bi_1.08_Sn_0.02_Sb_0.9_Te_2_S. It was prepared by the vertical Bridgman method (VBT). It also maintained the excellent properties of TI. For example, the carrier density was below 3 × 10^−14^ cm^−3^ at low temperature, and the bulk band gap was 0.35 eV with a Dirac point in it [106]. Figure 7 is the schematic of its QL structure, band gap and Dirac point. The color of the Bi/Sb atoms in Figure 7a was obtained from the reconstructed constituent units. The red line in Figure 7b represents the minimum value of the spectrum, and the brown line represents the average spectrum along the line direction. The arrow points to the approximate position of the valence band and the edge of the conduction band (BVB and BCB). The marked part with a gray dotted line is the bulk band gap (as shown in Figure 7c). The two crossed red lines correspond to the Dirac cone, and the green line shows Fermi level. Several key points of the Sn-BSTS low-band structure are shown in Figure 7d,e. Figure 7g–i is the Fermi level compared to the original sample. It can be seen from the figure that the band gap was similar to the common 3D TI such as Bi_2_Se_3_. The Dirac point was closer to the valence band indicating that it had the characteristics of the TI. However, there are no follow-up reports.

(6) Cd: In 2011, Ren et al. fabricated p-type Cd_0.002_Bi_1.998_Se_3_ in Se-rich environment and controlled the increase of Se vacancies to achieve p-n type conversion [93]. It should be noted that Wang et al. also achieved the conversion of the carrier type by controlling the annealing temperature in the same year. In contrast, TI was not doped [66]. So it is hard to determine whether the Cd doping led to the change of carrier type. 

(7) Ca: In 2011, Hor et al. studied Ca-doped Bi_2_Se_3_ and found that the Fermi level of Ca_x_Bi_2−x_Se_3_ was moved into the band gap. The carrier type was changed from n-type to p-type [101]. Moreover, Ca impurity could change the Hall density symbol by adjusting gate voltage, thereby reducing the bulk carrier concentration [107]. This is potential to the practical application.

(8) Ir: There is less research on Ir impurity because Ir is radioactive. Ir-doped TI still retains the properties and structure of TIs [97]. When the doping amount was low (x ≤ 0.05), Ir_x_A_1−x_B_3_ was the main doping method, just like Fe impurity. However, Ir_x_A_2_B_3_ became the main chemical formula with the increase of x (x = 0.07) [108]. Magnetic moment and resistivity showed a tendency to decrease, while the lattice constant c had the opposite trend. It can be inferred that these properties had a connection with doping type.

It can be found from the above discussion that doping has a great effect on the properties of TI including its carrier type, magnetic and electrical properties. It should be noted that scientists from different countries are interested in the superconductivity of TI. The superconductivity also can be observed at a certain transition temperature by different impurities. 

Table 3 compares data for the superconductivity of doped TIs in recent years. Some preparation methods are mentioned in it—modified bridgman method (MBM), melt-growth method (MGM), modified floating-zone method (TSFZ), floating-zone method (FZ), vapor transport method (VTM) and solid-state-reaction method (STR). These are common preparation methods used in the laboratory. Table 3 shows that the study of In impurity is the highest. The different doping amount also corresponded to different transition temperature. In addition, the change of pressure led to the change of transition temperature. It should be noted that different transition temperatures were observed with same x. The reason for this may be as follows. (1) The preparation methods were different. The properties of the material were slightly different by unique methods. (2) Experimental equipment and conditions were different. Superconductivity change was sensitive so the experimental conditions will also make the results have some errors. Furthermore, it can be found from the data that the research on the superconductivity of TIs has been extended to the TCIs, especially the study of In and Cu impurities. The Pd-doped TI had the highest transition temperature, while the transition temperature of In-doped TI was the lowest.

#### 3.2.2. Magnetic Impurities

The time-reversal symmetry on the TI surface will be destroyed by the magnetic impurities. The band gap of the surface will also be opened resulting in the loss of the perfect metallic surface. Then some special physical phenomena can be observed [49,121,122]. The main magnetic elements are Fe, Mn, Cr, Co and V, and so on.

(1) Fe: In general, Fe_x_A_2−x_B_3_ is prepared by mixing high purity Fe powder with Bi powder and excess Se powder (for reducing Se vacancy defect). Annealing treatment was required at 550 °C for a long time, which was helpful for dispersion of Fe atoms into the A_2_B_3_ crystal lattice. The carrier concentration was also reduced by an order of magnitude [97]. After doping Fe impurity, the lattice parameters of the TI showed a tendency of decrease first and then increase. This was mainly due to the atomic radius of Fe (1.27 Å) being smaller than that of Bi (1.70 Å). However, with the increase of x, the dopant was close to saturation. Then more Fe atoms were inserted between QL-QL (Fe_x_A_2_B_3_). Also, the crystallinity of single crystal also decreased. Different x had great influence on the electron mobility of the TI (from 300~2500 cm^2^/V·s) [97,123]. Furthermore, its resistivity increased nonlinearly, and its magnetic quantization became stronger [124].

**Figure 8:**
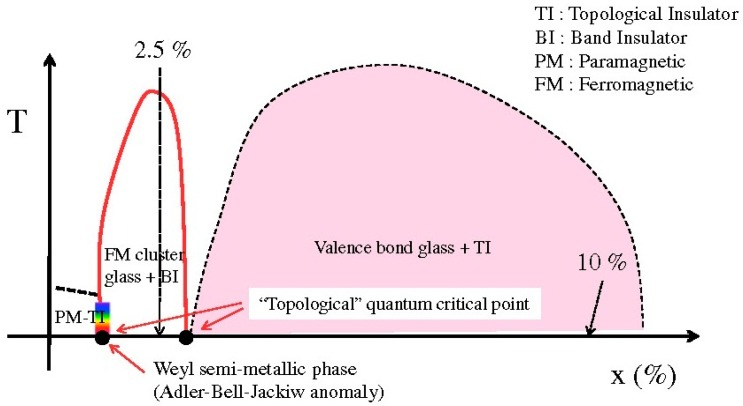
(Color online) Phase diagram and topological phase transitions of Fe_x_Bi_2_Te_3_ [125].

In 2013, Kim et al. found that Fe_0.025_Bi_2_Te_3_ changed from ferromagnetic to antiferromagnetic phase, and turned into a band insulator with ferromagnetic-cluster glassy behavior [125]. Figure 8 shows the phase diagram and topological phase transitions of Fe_x_Bi_2_Te_3_. It can be seen from the figure that the Curie-Weiss temperature reached its peak at x = 0.025. Ferromagnetic-cluster glassy behavior, magnetic resistance and Hall effect dramatically changed. It opened a gap in the Dirac point of the surface band. 

**Figure 9:**
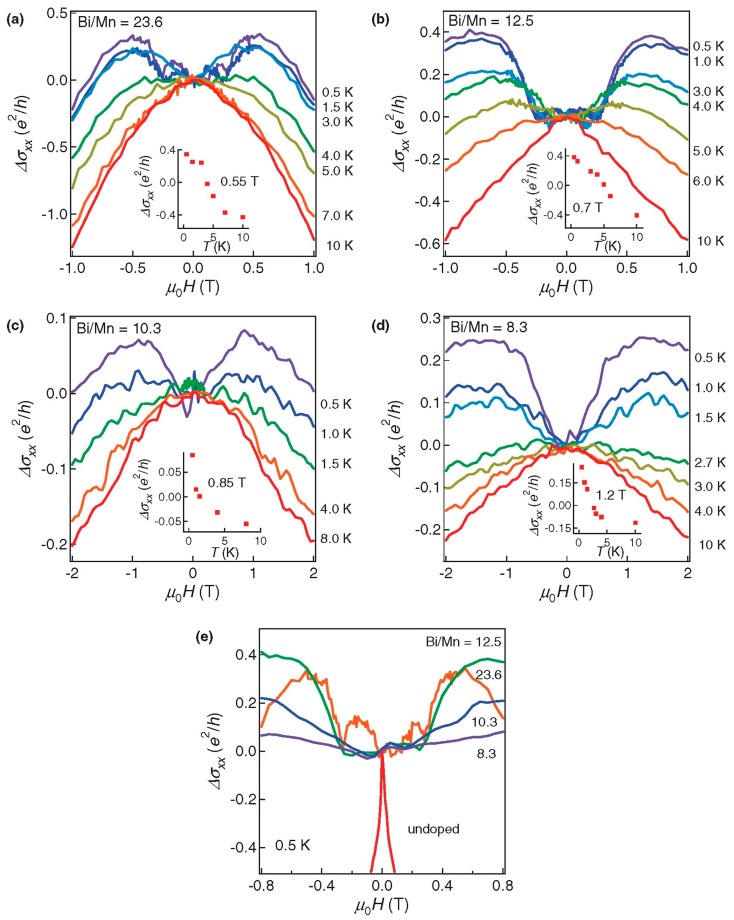
(Color online) MC of Mn-Bi_2_Se_3_ in field perpendicular to the sample plane: (**a**) MC of the most lightly doped sample (Bi/Mn = 23.6); (**b**) MC of the Bi/Mn = 12.5 sample; (**c**) MC of the Bi/Mn = 10.3 sample; (**d**) MC of the most highly doped sample (Bi/Mn = 8.3); (**e**) MC of four Mn-Bi_2_Se_3_ and one undoped Bi_2_Se_3_ sample [126].

(2) Mn: The Mn_x_A_2−x_B_3_ was the main chemical formula of Mn-doped TI. Mn impurity had great influence on the electromagnetic transport properties of Bi_2_Te_3_ and Sb_2_Te_3_. In actual preparation, the amount of Mn dopant was not easily controlled. In 2012, Zhang et al. prepared the Mn-doped Bi_2_Se_3_ thin film by MBE. However, the distribution of Mn atoms were uneven so the quality of the film was not very good [126]. The structure, magnetic, electronic and spectral properties were investigated (15 K < T < 100 K). Figure 9 shows the effect of ferromagnetism on the magneto-conductivity (MC). It can be seen that at high temperatures, all samples showed the negative MC and then exceeded the positive MC as the decrease of temperature. In contrast, the MC of undoped Bi_2_Se_3_ was always dominated by a characteristic cusp-like negative MC in the low field.

In 2010, Hor et al. predicted that Mn_x_Bi_2−x_Te_3_ might have a thin ferromagnetic semiconducting characteristic [127]. In addition, controlling the amount of Mn impurity could regulate the surface state of the TI. However, the principle was not deeply discussed. Niu et al. successfully used the first-principles to calculate the effect of Mn dopant on the electromagnetic transport properties of Bi_2_Te_3_ thin films next year [128]. The results showed that Mn impurity can induce the spin-polarized hole states (the total magnetic moment is 4.068 μB). The sufficient hole carrier density was required to achieve sustained magnetization. In 2010, Chen et al. observed the Mn-doped TI, but did not find any ferromagnetic origin. Moreover, when the Mn amount was more than 1.25%, it was a p-type semiconductor [129]. It can be seen that the magnetic effect of Mn on the TI still had divergence. 

The micro-morphology of Bi_2_Se_3_ was changed by Mn doping. The edge of the Mn_x_Bi_2−x_Te_3_ sample had an epitaxial trend, which was likely to cause dramatic changes in electrical properties. Bi_2_Se_3_ was an n-type semiconductor with a conductive property (x < 0.05), but the Fermi level shifted below the Dirac point as x increased. Then it changed into the p-type semiconductor exhibiting semiconductor characteristic. By systematically controlling Mn amount, it was also possible to control the dynamics of spin-Dirac fermions [18]. In addition, Mn_x_A_2−x_B_3_ exhibited paramagnetism. There were no hysteresis loops at low temperatures because of the presence of antiferromagnetic interaction among Mn^2+^.

(3) Cr: In 2015, Mogi et al. doped 1 nm thick Cr_0.46_ (Bi_0.22_Sb_0.78_)_2_Te_3_ thin film to the 1 nm upper and lower surfaces of the (Bi_0.22_Sb_0.78_)_2_Te_3_ thin film. The observable temperature of the QAHE was increased from 300 mK to 2 K [130]. Cr impurity had a great influence on the magnetic properties of TIs. Cr-doped films could achieve magnetization conversion of spin-orbit torques (SOTs). Although the SOTs were large, they can still be controlled. In the same year, Fan et al. proposed a top-gate FET structure of the Cr-doped TI, which could achieve the control of the electric field. The range of the gate voltage increased fourfold. This could reduce the power dissipation of TI, which could apply it to SOTs equipment [131].

## 4. Application of TIs

Chapters 1 to 3 have discussed the development, characteristics, preparation methods and doping of TIs. This chapter mainly introduces the application of TIs in practice. In general, the emergence of new material will attract scientists to promote the study of it. TIs not only shocked condensed matter physics, but also extended some new application fields. The ideal TI system has high surface electron mobility. However, the TI inevitably has some defects due to the constraints of preparation method and process. This makes the actual parameter lower than the theoretical value and thus limits its practical application. In view of this problem, many scientists have carried out research on it. They found that high-purity single crystal TI could be fabricated by optimizing the preparation process and using high precision equipment. The properties can also be improved by doping.

Research on TIs mainly remains in the theoretical stage. A large number of papers that study the intrinsic properties of TIs have been reported. They are mainly concerned with the calculation of the first principle, the TI surface state, QSH and superconductivity. In contrast, there are relatively few studies on the application of TIs so many applications do not achieve the desired effect. The actual performance of TI-based devices does not reach the theoretical value. The TIs have many unique and excellent properties including optical, electrical and magnetic properties. In addition, the narrow band gap and the QL structure make them have high application and research value. It can be seen from the discussion in chapter 1 to 3 that there are three main points for the study of TIs: (1) Exploring and predicting the ideal TI such as the TCIs in recent years; (2) Theoretical study on the physical issue of TI by condensed matter physics; (3) The practical application of TIs. At present, the dominant applications are some electronic and semiconductor devices such as photodetectors, magnetic devices, FETs and lasers. This chapter will discuss these TI-based devices.

### 4.1. Photodetector

Photodetectors are widely used in military, medical, testing and other industries. The light-sensitive material is irradiated to cause the change of its conductivity. The different wavelengths of radiation correspond to different conductivity, which can be used to detect diverse wavelengths of light. That means that the photodetector can convert the optical signal into the electrical signal. Photodetectors are divided into two categories: thermal detectors and photon detectors. The former mainly works through the absorption of incident radiation and heat, while the latter directly absorbs the photons. Photodetectors have some important parameters including responsivity (R), specific detectivity (D*), response time (rise time t_r_, fall time t_d_), equivalent noise power (NEP) and quantum efficiency (η).

The graphene has a broad band of absorption spectrum, but graphene is a monolayer material with no band gap. After several years of development, the responsivity of the graphene-based photodetector has risen from mA·W^−1^ to A·W^−1^ [132,133]. The band gap of the TI is narrow, and the crystal is a QL structure similar to that of graphene. The TI film also has good transparency and wide absorption spectrum, so it is a good photoelectric material. The TI-based photodetector has high responsivity, fast response time and wide band (UV to near infrared). Narrow band gap also improves its conductivity.

**Figure 10:**
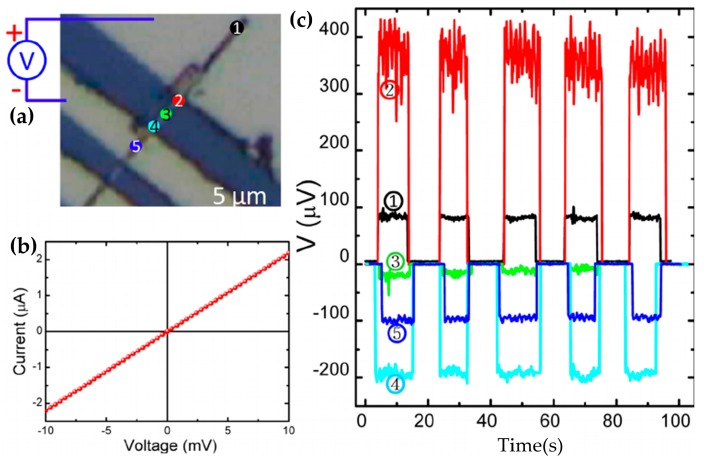
Photovoltage generation from a single Bi_2_Se_3_ nanoribbon device with vertically incident linearly polarized light: (**a**) Typical optical image of a Bi_2_Se_3_ nanoribbon device; (**b**) I−V curve of a typical Bi_2_Se_3_ nanoribbon device; (**c**) Photovoltage response with switching on/off the laser illumination [134].

In 2014, Yan et al. applied the Bi_2_Se_3_ nanoribbons synthesized by CVD to photodetector and used the topological surface to enhance its performance [134]. In the circularly polarized light (CPL), the topological surface state was spin-polarized, resulting in a photocurrent. The CPL excited the topological surface state to produce additional electrons whose direction of motion was the same as that of the temperature gradient. Therefore, the oriented motions of electron spin were constantly accelerated to produce high voltage (max 400 μV). According to the working principle, this device is a thermal detector. Figure 10 shows the test results for the device. This test used vertical incident polarized light (wavelength: 514 nm, spot size: 1 μm, power: 0.15 mW). Figure 10a indicates that the light was irradiated at different positions on the device. The red symbol “±” represented the positive/ground electrode. The numbered circles marked the irradiation position. Figure 10b shows the current-voltage relationship in the absence of light, which suggests that the device had good ohmic contact. Figure 10c shows the corresponding voltage. It indicates that the device had different generated voltage at various positions. The diverse curves corresponded to the illuminated positions marked in Figure 10a.

The TI surface state is protected by time-reversal symmetry so it can be prevented from scattering by other non-magnetic impurities. Also, its ability to absorb light was excellent. Even infrared or ultraviolet could also be absorbed [135]. In 2015, Yao et al. confirmed this [136]. They used Bi_2_Te_3_-Si heterostructure thin film to produce a photodetector with ultra-wide band (ultraviolet to terahertz) and high responsivity working at room temperature. In addition, the stability of the device was good. This means that it still kept the parameters basically unchanged after exposing to the air or strong light for a long time. Under the built-in electric field, the photo-generated carriers were separated. These carriers could be collected quickly because of the high surface mobility of the TIs. Thus, the response time was greatly reduced. Furthermore, the TI-based photodetector could work without bias because of its low dark current, high sensitivity and detection and low energy dissipation. In the same year, Zhang et al. proposed a photodetector with much higher responsivity than that of J. Yao’s study. However, its band, response time and detectivity were not as high as Bi_2_Te_3_-Si heterojunction photodetector [137]. In 2016, Yao et al. continued the study of TI heterojunction [138]. They used PLD to deposit WS_2_/Bi_2_Te_3_/SiO_2_ films on Si substrates. The responsivity of WS_2_/Bi_2_Te_3_ heterojunction photodetector was 30.7 A·W^−1^, which was significantly higher than that of Bi_2_Te_3_-Si heterojunction photodetector (1 A·W^−1^). In contrast, the detection range was not up to terahertz. The reason for this might be that the sandwich structure limited its band. Zhang et al. fabricated a Bi_2_Te_3_-Si heterojunction photodetector with high detectivity, which was one order of magnitude higher than that described above (4.39 × 10^12^ cm·Hz^1/2^·W^−1^). The response time also reached the microsecond level [139]. It is worth mentioning that the PVD method was used to fabricate the photodetector so the cost was relatively low. SnTe, as the third-generation TI, is used in photodetectors. Jiang et al. utilized the MBE to deposit 4 nm Bi_2_Te_3_ on the Si substrate and then deposited 10 nm SnTe to obtain high-quality SnTe thin films. This is because the lattice constant of Si or strontium titanate (STO) did not match SnTe, thereby producing 3D rather than 2D structure. The SnTe-based photodetector had high band (405 nm–3.8 μm), but other properties were not superior to the 3D TI-based photodetectors [140].

Table 4 compares the parameters of the different TI-based photodetectors. Some related parameters of graphene photodetector are also listed in Table 4 as the graphene and TIs are popular semiconductor materials, and they are also similar in structure. This can help to comprehensively compare the advantages and disadvantages of the two photodetectors. There are some preparation methods (PM) in Table 4: mechanical exfoliation (MEM), hydrothermal method (HTM) and layer by layer transfer method (LBLT). The common structures of TIs-based photodetectors are nanowires (NWs) and nanoplatelets (NP), etc. The structures of graphene are monolayer (ML) and bilayer (BL).

As can be seen from Table 4, although the research on TI-based photodetector was late, its performance was excellent, especially the band. It reached the terahertz. The band range basically covered from ultraviolet to visible and then to infrared. Another advantage of TI-based photodetectors was that the responsivity was high. It could achieve the order of A·W^−1^ (up to 1000 A·W^−1^). It should be noted that the composite structure of graphene and colloidal quantum dots (QDs) could improve its responsivity. Therefore, the combination of TIs with QDs might greatly improve the responsivity. The detectivity of TI-based photodetectors were also 3 to 4 orders of magnitude higher than that of graphene photodetectors, which suggested that the weak signals could also be captured. It can also be seen from Table 4 that heterogeneous structure was widely used in graphene, graphene oxide (GO) and TIs photodetectors such as graphene-MoTe_2_-graphen (GMG). This p-n junction could greatly improve the responsivity and response time of the photodetectors. In the case of heterogeneous structure, the response time could remain at the ms or μs level so that it could be applied to the fast response devices. Furthermore, it could absorb UV to near infrared light (NIR) since the band gap of the TI is narrow. The electron-hole pairs were generated at the heterojunction. After that, the photocurrent was generated by separating the electron-hole pairs to the two electrodes under the action of a built-in electric field. In addition, the built-in electric field and heterojunction could also prevent the electron-hole pairs from recombining. This is the reason why the response time of the TI-based photodetector was better than other photodetectors. The detectivity was enhanced by the low dark current.

There were cases when graphene was combined with TIs, but most of them were second-generation TIs. If the third-generation TI could be combined with graphene, it might create devices with better performance. The preparation method, structure and substrate also had an influence on the photodetectors. At present, the CVD or PLD method are commonly used, which can obtain high-quality crystal structure. However, their cost was high so they were only suitable for research. Of course, there were cases of using PVD, which could also get excellent performance. The preparation method had a relationship with the structure of TIs. The film and layered structure were mainly used. Also, the substrate had an influence on the performance of device. It is suggested to choose Si or SiO_2_ substrate with lower resistance.

Overall, although TIs has been used in photodetectors, there are still some problems. The ideal TI has high surface electron mobility, but its performance does not currently reach the theoretical value. The main reason for this is because of the material defects including intrinsic defects and doping defects. However, with the research of TIs, these problems are likely to be solved.

### 4.2. Magnetic Device

The TI has strong QSH, and its surface has a spin-related conductive channel. This means that the non-zero spin density flow spontaneously appears when the electrons flow on a TI surface. If the ferromagnet (FM) and the TI are coupled to form a heterojunction, the surface current can be used to control the FM, thus developing a new type of spin moment and magnetoresistive devices. For example, magnetic tunnel junctions (MTJs) can be used to study the tunneling magnetoresistance (TMR). The spin-orbit coupling surface state of the TI can enhance the spin-related tunneling effect. Only the weak external magnetic field can reverse the magnetization direction of one ferromagnetic layer, hence a huge change in TMR is achieved. As described in Chapter 3, doping can make the TI exhibit many novel physical phenomena such as magnetic monopole. In addition, some magnetic impurities will destroy the surface state of TI and open a band gap. Also, the ferromagnetism is even converted into antiferromagnetism. In a word, the TI has prospect in low energy dissipation, magnetic transport and magnetoresistance fields.

**Figure 11:**
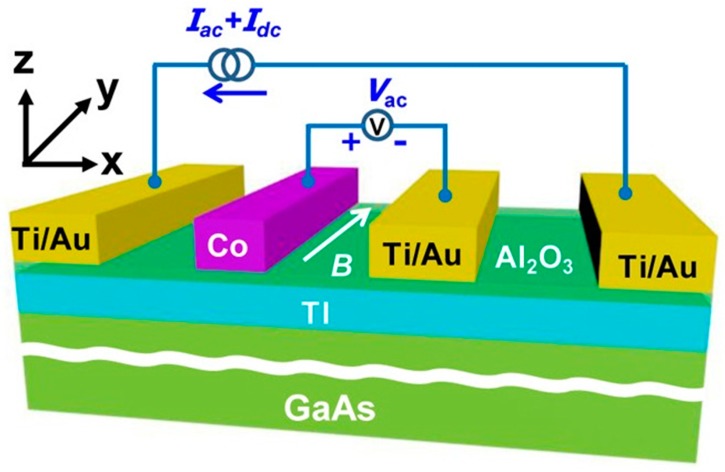
Schematic illustration of the device structure with one ferromagnetic tunneling Co/Al_2_O_3_ contact for spin detection [148].

The spin-momentum locking of the TI surface contributes to the development of spin electronic devices. It is also potential to achieve dissipationless transport. In 2014, Tang et al. fabricated ferromagnetic tunneling device combining (Bi_0.53_Sb_0.47_)_2_Te_3_ thin film with Co/Al_2_O_3_. The resistance of hysteresis amplitude reached 10 Ω [148]. They did not directly use the second-generation TI to make device because the hole doping was likely to exist during the process of preparation. It resulted in higher carrier density, thus affecting the performance of the device. Figure 11 shows the schematic illustration of the device. The Ti/Au was non-magnetic contact mainly used to conduct the electricity. In contrast, the Co/Al_2_O_3_ was a ferromagnetic tunnel contact used to detect the spin polarization of the TI surface state. In addition, the (Bi_0.53_Sb_0.47_)_2_Te_3_ was grown on the GaAs substrate by the MBE. The working principle of the device was to change the magnetization orientation of the Co electrode by altering the magnetic field. Then the polarization direction of the electrons was also changed on topological surface, thus casing hysteresis. When the Co magnetization and the surface spin polarization direction were opposite, it showed a low resistance state. Otherwise it showed a high resistance state. In 2015, Tian et al. completed the spin potentiometric measurements of Bi_2_Te_2_Se [149]. The structure of the device was similar to that of Tang et al. The spin current was also driven by non-magnetic contact, and the ferromagnetic Al_2_O_3_ contact was used to detect the surface polarization current. Furthermore, the ladder-like voltage hysteresis appeared while scanning the magnetic field.

Although the spin-polarized surface of the second-generation TI could be detected by conventional accumulation voltage measurement, the TI was often required to prepare thin film. This limited its prospects. Therefore, the TI compounds were fabricated by synthetic method. For example, the Bi_1.5_Sb_0.5_Te_1.7_Se_1.3_ (BSTS) was prepared by the Bridgman method. In addition, the Ni_80_Fe_20_ was used as a ferromagnetic electrode. The reluctance of the BSTS was related to the direction of the current, and a spin-dependent resistance appeared at the junction of the electrode and the BSTS. Ando et al. found that the magnetoresistance could be changed by controlling the current direction or the magnetization direction of Ni_80_Fe_20_ at the temperature of 4.2 K [150]. In general, the spin polarization current of the TI surface could be detected by magnetoresistance measurement or accumulation voltage measurements. However, the magnetoresistance measurement was only applicable to BSTS rather than other second- generation TIs. Furthermore, the magnetoresistance at the junction of the BSTS and electrode could not be controlled at high temperatures (300 K or more). For this reason, this method had limitations.

Conventional TMR devices had a ferromagnet/insulator (tunnel barrier)/ferromagnet (F/I/F) sandwich structure. In contrast, TIs could not only be applied to both traditional F/I/F structures, but we also observed spin-related tunneling effects in some new structures. For the first case, the conductivity of the tunnel junction could be controlled by changing the magnetization direction of the two ferromagnetic sections. Also, the magnetoresistance could be maximized by adjusting the magnetic gap. The recommended thickness of TI was 3–5 nm, which had the highest degree of coupling [151,152]. There have been reports on the preparation and characterization of FM such as GdN and Bi_2_Se_3_ heterojunctions with low bulk carrier density. The FM/TI heterojunction exhibited a magnetoelectric effect that had electrical modulation and anisotropy. This formed the basis for the development of heterojunction and low dissipation devices [153,154,155]. For the second case, improvements could be made on the basis of the traditional structure, thereby greatly improving the TMR ratio. For example, Bi_2_Se_3_ was applied to the TMR device eliminating the second layer of FM. In contrast, a U-shaped FM/insulator/TI structure was used to improve the TMR ratio exceeding 490% [156]. It should be noted that if the device was made into a 2D structure, the TMR ratio could even exceed 1000%. Furthermore, it is possible to deposit two FMs on the surface of TI and form a deformed sandwich structure. It could also control the reluctance by adjusting the spin polarization current [157].

It is worth noting that not only 2D and 3D TIs could form tunnel junctions, 1D topological superconductors and TI (integer) could also be used to fabricate tunnel junctions. However, special methods were needed—bosonization and renormalization group methods [158]. The interface was divided into the two parts. One side had strong coupling Majorana fermions showing Andreev reflection, while the other side only showed normal reflection. This novel phenomenon could be used to study Majorana fermions, but it only had theoretical and experimental value such as quantum computing. This point contact was hard to apply to the device. Moreover, FM/TI/FM was widely used to study the TMR effect. It could also be applied to the thermoelectric switch spin device [159]. With the study of the TIs, the tunnel junction structure would be more widely used in the magnetic and spin devices field.

In recent years, there have been more studies on s-wave and p-wave superconductors. When the total electron spin is zero, it is called the s-wave superconductor, while the p-wave superconductor is the case that the total electron spin is 1. It was similar to the FM/TI/FM tunnel structure, a ferromagnet (/insulator)/superconductor (FM/I/SC) structure was established on TI surfaces. Its tunnel conductance could be studied using Blonder-Tinkham-Klapwijk (BTK) theory. In general, the main factors included magnetic gap, gate potential and quasiparticle lifetime [160]. Furthermore, it is worth noting that the FM/I/SC could also be established on the surface of the graphene, but its electrical properties were different from those of the TI. Although they had similar Hamiltonian, the spin-orbit coupling polarity was opposite. The graphene was weak spin-coupled, while TI was strong spin-coupled. TI surface also had a special magnetic and superconductivity [161,162,163,164].

In conclusion, due to the unique surface state of the TI, it has a wide range of applications in magnetic devices. However, now its magnetic research is still in the theoretical and experimental stage. The research on TI and FM tunnel junction attracts many scientists. It is also a promising filed.

### 4.3. Field-Effect Transistor

FET is a kind of voltage-controlled semiconductor device. It has many advantages—high input resistance, low noise, low power dissipation, large dynamic range, easy integration, no secondary breakdown, wide working range, and so on. It is also known as a unipolar transistor because the majority of carriers are involved in conduction. The drain current (IDS) flows through the channel between the drain and the source. It is controlled by the reverse bias gate voltage formed by the p-n junction between the gate and the channel. FET is mainly divided into junction FET (JFET) and metal-oxide semiconductor FET (MOS-FET). MOS-FET is now widely used. FETs have gate, drain and source. The input current of the FET is low (approximate 0). It is widely used in large-scale and ultra-large scale integrated circuits because of its advantages. The electrons on TI surface can pass through the band gap and the motion directions of different spin electrons are also diverse. Therefore, it is beneficial to make low-power transistor devices.

**Figure 12:**
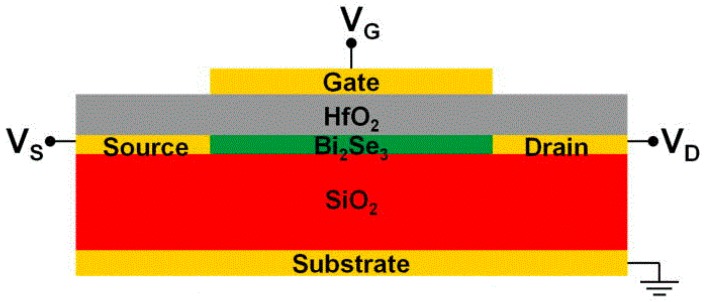
Device structure of Bi2Se3 metal-oxide semiconductor-field-effect transistor (MOS-FET). The nominal device parameters are as follows: Bi_2_Se_3_ (*κ* = 100) thin film = 1QL (~0.7 nm), HfO_2_ (*κ* = 25) gate oxide thickness = 2 nm, channel length = 20 and 50 nm, n-type doping density of source and drain = 1 × 10^13^ cm^−2^ [165].

TIs have perfect metallic surface and narrow band gap. In practice, TI was often prepared into the thin film. The thickness of the film determined the size of the band gap. When the Bi_2_Se_3_ film was thicker than 6QL, it had metallic surface and 0.3 eV band gap. However, when the thickness was less than 6QL, the surface band gap was not zero due to the interaction between the conduction band and the valence band on the upper and lower surface. Its band gap decreased as the thickness rose. Furthermore, the Bi_2_Se_3_ film could be used as channel material for MOS-FETs. Figure 12 is the schematic diagram of an MOS-FET structure [165]. The IDS reached the maximum of 1.1 mA/μm at gate-source voltage (VGS) = 0.7 V when the channel length was 50 nm. The ratio of the maximum (VGS = 0.7 V) to the minimum current (VGS = −0.6 V) was about 1012. Bi_2_Se_3_ had high dielectric constant so its potential changed slowly. This resulted in poor performance when the channel length was 20 nm, but this could suppress the drain leakage caused by the gate. Compared to 1QL Bi_2_Se_3_-based MOS-FETs, the performance of conventional Si-based MOS-FETs seemed to be better. In contrast, this was only the use of Bi_2_Se_3_ with high dielectric constant for FETs. The TIs with lower dielectric constants also had potential, e.g., stannanane, a TI with monolayer hexagonal structure [166]. Stannanane (functionalized with iodine) was applied to the FET. Its maximum current of 10^4^ A/μm could be obtained at drain-source voltage (Vds) = 0.25 V. The on/off current ratio (Ion/off) stood at about 104. These parameters were equivalent to or better than the traditional high current Si-FET. What is more, the energy dissipation of TI-FET was lower. If Bi_2_Se_3_ was prepared as nanowire and used as the conductive channel for FET, it could increase its Ion/off up to 108 [167]. In addition, increasing the number of layers of QL could also improve the performance of TI-FET (film) [168]. It is possible to get more excellent TI-MOS-FET with further study.

One of the advantages of TI-FET is low energy dissipation. This advantage can be highlighted by designing a new structure of the FET. S.K. Banerjee et al. designed a new structure based on TI-FET. The two gate dielectric layers were separated by TI, and the upper and lower gate surfaces were connected to the two dielectric layers, respectively. This was referred to as gate contact [169]. It was transported by tunneling with the TI surface. This reduced the power dissipation while improving its logic voltage. Furthermore, some materials such as Al_2_O_3_ could be deposited directly on Bi_2_Se_3_ so that this composite could be applied to the FETs. About 76% of the modulation rate could be reached. Moreover, the Bi_2_Se_3_-FET with ionic liquid gate could even achieve 365% modulation rate [170,171,172]. However, more excellent TI-FET would be fabricated if the high-quality TI could be prepared. It should strictly control the position of Fermi level between the valence band and the conduction band. This means that the performance of the TI-FET would be greatly affected if the Fermi level was in the valence band or conduction band. Mehran et al. proposed that the Fermi level could be controlled through the ferromagnetic tunnel junction (FMTJ), which the Ion/off reached 104 [173]. The FMTJ structure has been discussed in detail in Section 4.2 so it is not described here. The preparation of TI has been a popular research direction. There are a large number of articles about the preparation and regulation of TIs. Therefore, it is likely to prepare large-scale and high-quality TI in the future, so that high-performance and energy-dissipation TI-FET could be industrialization.

The conventional FET generally uses Si or its compounds as material. In recent years, the rise of graphene has also led to the study of FETs. In order to better compare the advantages and disadvantages among different FETs, Table 5 is given below. The phosphosilicate glass (PSG) and lateral plasma etching (LPE) are mentioned in the table. Table 5 compares the performance of FETs based on TI, graphene and Si. It can be seen from the Table 5 that the Ion/off of Bi_2_Se_3_-Nanowire was the largest (108), which was about an order of magnitude higher than other FETs [167]. WSe_2_-hBN and Ge/Si Heterojunction also exhibited high Ion/off (107) [174]. Also, the IDS max of TI-FETs had high parameters, which were substantially equivalent or superior to other FETs. The data illustrates that the TI-FET had good performance. The reason for this was that TI has a perfect metallic surface and the appropriate band gap. As a result, TI-FETs can maintain excellent parameters while saving energy. This provides opportunity to fabricate large-scale production of efficient, energy-saving and stable FET.

### 4.4. Laser

The application of laser covers a wide range fields including optoelectronics science and other core technologies. Semiconductor lasers have been widely used in materials processing, precision optics, laser ranging and medical field. Most lasers require saturable absorber (SA) because they are based on passively mode-locked or Q-switched mechanisms. The SAs are divided into artificial and real materials. Compared with the artificial saturable absorber mirror, the real SAs (graphene, WS_2_, TI, etc.) have wide wavelength, simple preparation, low cost and wide application value. The TI, as excellent semiconductor, can be used in SA like graphene. This is because the surface state of the TI has linear dispersion relation. It exhibits a nonlinear relationship under the excitation of high energy pulses and has linear relationship at low energy. According to the requirements of a device, TI's thickness was generally nano- to micro- scale [185]. TI could be directly deposited on the fiber. Also, TI nanoplate solution filled with photonic crystal fiber could be used as SA and thereby reducing the insertion losses [186,187].

Due to the limitations of the soliton area theorem, it is generally difficult to reduce the pulse duration to less than 200 fs in the absence of the dispersion compensation mechanism. In addition, if the average nonlinear parameter was too high, the peak power of the soliton in a passively fiber laser operating in anomalous dispersion regime was difficult to improve [188,189]. In order to solve the traditional pulse energy limit, it was necessary to balance the dispersion and nonlinearity by gain and loss equilibrium [190]. Therefore, the dissipative soliton operation was important. Lee et al. used Bi_2_Te_3_ as SA to fabricate a laser with pulse duration of 600 fs [185]. In the same year, Sotor et al. deposited Sb_2_Te_3_ (0.5 mm thick) on the laterally polished erbium-doped fiber and successfully made a passively mode-locked laser with pulse duration of less than 200 fs (128 fs) using a dispersion compensation fiber [191]. Its modulation depth (ΔT) was 6%, and non-saturation loss was 43%. Although the structure of the two lasers was similar, the latter used the dispersion compensation to reduce the pulse duration. The ΔT was also half as high as the former (16%). It should be noted that if the pulse duration of the mode-locked laser was to be stabilized at the femtosecond level, the ΔT could not be too high. The reason for this was that with the increase of the ΔT, the stability of the gain saturation would be reduced, which was likely to cause passively Q-switched laser. 

Table 6 is the data for different mode-locked lasers. In general, the TI was deposited on the side-polished fibers resulting in the interaction between the evanescent fields of propagating beams. In addition, TI could be combined with other materials such as Bi_2_Se_3_/Polyvinyl Alcohol (PVA). The mode-locked lasers working in two wavelengths could be produced by adjusting the pump power and polarization state. Furthermore, the wavelength range was likely to be affected by changing the length of the fiber [192,193,194]. However, its pulse duration reached the magnitude of ns, which was significantly lower than that of other mode-locked lasers.

Table 6 shows that the minimum time duration of TI-based mode-locked lasers stood at 70 fs [196]. The ΔT of the mode-locked lasers was small (about less than 10%), which might to keep lasers stable. Furthermore, the highest repetition rate was up to 2950 MHz [209]. Lasers with high repetition rate could be used for telecommunications, spectroscopy and metrology. It was found that the different carrier types of TI also changed its parameters, but the magnitude was not significant [214]. The saturating intensity (I_sat_) of the most TI-mode-locked lasers was less than 20 MW/cm^2^. In general, the S/N should be no less than 70 dB, but that of TI laser was generally lower than this value (maximum value was 77 dB), which needed to be improved [207]. Another advantage of TI-based lasers was the wide wavelength (from 800 nm to 1935 nm) [213,221]. However, most of the TI-based lasers operated at the wavelength of about 1500 nm, which was the common value. In addition to TI, some common materials such as MoS_2_, WS_2_, WTe_2_ and graphene were also extensively studied in the field of lasers. Some examples of excellent parameters were listed in Table 6. It can be seen from the table that the pulse duration of graphene-based laser reached 19 fs [229]. However, the S/N was not as high as TI-based lasers. WTe_2_-based laser’s wavelength could reach to 2970 nm, while the pulse duration and repetition rate were relatively poor [228]. In summary, TI is indeed a very promising SA material.

An active Q-switched laser requires the electrooptic modulator, which increases the complexity and cost of device. Passively Q-switched laser is compact, simple and versatile. It is generally applicable to medical, military, detection and many other fields. It also requires the SA like mode-locked laser. The frequency of SA could also be selected since the absorption cross section of the SA and the emission cross section of the gain material were different at diverse wavelengths [231]. TI was not sensitive to wavelength change. This made it able to work in two wavelengths or even in a certain wavelength range under the same vibration mode [232]. There was a sandwich structure combined TI with piezoelectric transducer. It could achieve passively Q-switched and active mode-locked modes by adjusting the acousto-optical modulation and pump power [233]. Similarly, Bi_2_Te_3_ and polymethyl methacrylate polymer (PMMA) were used to form a sandwich structure. It could work at around 2800 nm wavelength. The maximum output power (P_out max_) was also greatly enhanced (maximum 856 mW) [234]. What is more, the max pulse energy (E_p max_) was 18300 nJ [235]. These parameters were higher than that of other lasers. In addition to the above two modes, there was a called Q-switched mode-locked mode. It was a technology simultaneously using Q-switched and mode-locked mode to achieve the transmission of ultra-short pulse. The P_out max_ was higher, which could be used for thermal processing [236,237].

Table 7 compares different Q-switched lasers based on TI and other common materials. The max ΔT of TI-based Q-switched lasers stood at 51.3% (as shown in Table 7) [255]. High ΔT was beneficial to suppress the wave-breaking effect and output high pulse energy. It also reduced the time duration. The range of I_sat_ was large. The crystal quality or oxidation led to the decrease of I_sat_, but the low I_sat_ was beneficial to the realization of Q-switching. The E_p max_ and P_out max_ were 18,300 nJ [213] and 856 mW [212] respectively. High-energy lasers could be used for processing materials, sensing, and communication. Furthermore, different fibers would also affect the performance of the laser. The common doping were erbium, ytterbium, Nd: YVO_4_, Nd: Lu_2_O_3_ and Nd: LiYF_4_ [231,238,240,247]. Moreover, Ho^3+^-doped ZBLAN fiber could work at mid-infrared (near 3000 nm) wavelength [255]. Compared with TI-based mode-locked lasers, Q-switched lasers had larger wavelength range (604 nm [244]–2979 nm [255]). The max repetition rate and pulse duration were 940 kHz [246] and 93 ns [247]. For more direct comparison, some common SA materials such as MoS_2_, WS_2_, MoSe_2_ and graphene were also listed in Table 7. It can be seen that the ΔT of the TI-based laser was higher compared to that of others, but I_sat_ was lower. In addition, TI-based lasers were similar or slightly better than other lasers in other parameters.

For passively Q-switched lasers, TI’s I_sat_ was low. This means that they had low threshold and high sensitivity. This was mainly due to TI’s narrow band gap and the Dirac fermions on the surface. In contrast, graphene lasers had lower saturation power for the mode-locked lasers. The reason for this was that when the thickness of graphene was thicker than 5 μm, the saturation absorption rate began to decrease. The evanescent field interaction rose, while the beam propagation loss decreased. The graphene-based laser with thin thickness had lower ΔT. The ΔT of TI-based lasers could reach 98% at most [217]. However, TI's electron relaxation time was longer than 300 fs, which suggests it was a relatively slow SA material. In response to this shortcoming, TI could be combined with other materials that had shorter relaxation time to form heterojunction. Moreover, Q-switched and mode-locked modes could be both achieved by adjusting the parameters of the composite. In summary, a TI-based laser has a wider effective bandwidth. Its efficiency and cost make it a promising material.

### 4.5. Other Applications

There are many researches on photodetectors, magnetic devices, FET and lasers. In addition to the above devices, TI can also be used in gas sensors, memory and other fields, but relatively little studies have been reported in these areas.

The gas sensor is a converter that converts the gas signal into the corresponding electrical signal. Due to the unique physical properties of the TI, the surface conduction channels were directly exposed to the gas, which exhibited different conductance (I/V) and polarization under different pressures, humidity and chemical environments [267]. So it has certain application prospects in the future. In addition, TIs can also be used in the field of batteries and memory. In 2017, Tian et al. reported that the current-driven TI surface strong electron spin polarization could persist at low temperature and zero current conditions [268]. The magnetically doped TI surface can be used as a memory cell. By changing the magnetization state, it is possible to write in memory, while reading out from memory depended on the quantum spin effect of TI [269]. TI such as PbTe/Pb_0.31_Sn_0.69_Te had excellent optical characteristics, which could be used to fabricate optically controlled quantum memory [270]. The advantages of TI memory were low energy dissipation and high quality factor [271]. The previously reported FM heterojunctions could also be used for the fabrication of memory [155]. In addition, the Bi_2_Se_3_ was applied to an organic polymer solar cell to achieve the maximum photoelectric conversion efficiency of 4.37%. This figure exceeded the efficiency of the device based on e-MoO_3_, and it was better than that of the device without the hole transport layer [272].

## 5. Analysis and Future Prospects

According to the summary and analysis from chapter 1 to chapter 4, there is no doubt that the TI will have extensive application and development in the future. Although TI has made great strides in the last decade, it still faces some challenges of the research and development. The ideal TI system has the characteristics of high surface electron mobility, narrow bulk gap and adjustable gate electric field. However, the surface electron mobility is lower than the theoretical value. The material defects lead to large bulk conductance. Furthermore, a considerable amount of time and effort has contributed to the exploration of the intrinsic properties of TI in recent years. It mainly focuses on the physical characteristics of the TI such as its QSH, thermoelectric effect and superconductivity. In contrast, the research on TI as a functional material for practical device has been very limited. The continuous improvement of various preparation methods improves the crystal quality of TI. Also, different magnetic and non-magnetic impurities give the TI many novel properties such as superconductivity, magnetic monopole and QAHE. Doping can also control the Fermi level, carrier type and carrier concentration of TI, which lays the foundation for the application of TI. With the further research on TI, there is a great opportunity to prepare the high-quality TI. It is also possible to achieve industrialization. In addition, more research on TI-based devices is needed to produce superior devices with better structure and properties.

On the one hand, we still need to explore the new generation of TIs with better nature, more stable structure and easier preparation process. Apart from classic TIs, advanced TCIs, DSMs and WSMs (type-I and type-II) have attracted the attention of scientists in recent years. The new theory and application of these new TIs also have great research value. It should be mentioned that the popular study involves the chiral anomaly of WSMs. However, the application of the new TIs still awaits further study. TCIs have potential applications in high-speed topological logic devices such that the crystalline symmetry can be controlled by using electrical field or strain because the mass of Dirac fermions is tunable by controlling mirror symmetry. It is very important to improve the preparation process and the regulation method because it is relatively difficult to prepare high-quality TI with narrow bulk band gap. In addition, the Fermi level is hard to control between the valence band and the conduction band. Moreover, research on the preparation of the different sizes of nanowires, nanoribbons and thin film is also needed. Another promising research field is the special nature of the TI studied through the precise control of defects and impurities (magnetic and non-magnetic). Different preparation methods have diverse characteristics. The most appropriate preparation method should be selected based on process requirements, accuracy, cost, product scale and structure. The superconductivity of TI has research value at low temperatures. Different transition temperatures can be obtained by different impurities and amounts. The advanced research is basically based on the third-generation of TI, and much attention is paid to In impurity. Magnetic impurity is helpful for the development of magnetic devices because it breaks the time-reversal symmetry on the surface of the TI.

On the other hand, TI, as a semiconductor, has excellent physical, chemical and photoelectric properties. It can be used in photodetectors, magnetic devices, FETs, lasers and other fields. In addition, it has also been used in the fields of gas sensors, quantum memories and batteries in recent years. However, there are only a few studies in these areas, and they are basically at the initial stage. The TI-based electronics exhibit good performance because of the novel surface state. The photo-generated separated carriers could be collected quickly in a TI-based photodetector because of the high surface mobility of the TIs under the built-in electric field. Also, The CPL can excite topological surface state to produce more Dirac electrons. The motion orientation is same as the temperature gradient, which constantly accelerates the oriented motions of electron spin to generate ultrahigh voltage. As for magnetic devices, the spin-orbit coupling surface state contributes to the spin-related tunneling effect so only a weak external magnetic field can cause significant change of TMR. Furthermore, the Dirac electrons on topological surface are capable of passing through the band gap and the motion orientations of different spin electrons are also diverse, which suggests that it is possible to make ultralow-power transistor devices. The surface states spin transfer torque also benefits the TI-FET. Moreover, the surface state of the TI has a linear dispersion relation. It shows a nonlinear relationship under the excitation of high energy pulses, while has a linear relationship at low energy, which meets the requirement of SA. Therefore, TI can be used to fabricate SA in mode-locked and Q-switched fiber lasers. 

Through the comparison in Chapter 4, it can be seen that the device based on TI is excellent. Photodetector with TI heterojunctions exhibits even more excellent properties. However, there is no report that the TI and the graphene are combined into heterojunctions as a photodetector. Therefore, the combination of TI and other materials is a promising research direction. It should be noted the photodetectors based on Bi_2_Se_3_, Bi_2_Te_3_ and other TIs still have the possibility to progress. This is related to the late discovery of TI. The substrate also has a certain effect on the photodetector. If it is further studied, an even more excellent photodetector will be fabricated. What is more, most magnetic devices of TI are based on an FM tunneling structure. However, the study of its magnetism is still in the theoretical and experimental stages. The TI-based FET has large I_on/off_ and I_DS_. The TI-based lasers are the most studied, which have excellent parameters as SA. It has the advantages of wide wavelength range, low saturation intensity, high repetition rate, large pulse energy and small time duration (fs and ps level). It is applied to Q-switched and mode-locked lasers. However, the signal-to-noise ratio of the TI-based laser is lower than 75 dB. Therefore, more effort is needed to improve the signal-to-noise ratio and stability of the laser. It is worth mentioning that the TI-based devices have the characteristics of low power dissipation because the electrons on the TI surface are not affected by the dispersion and non-magnetic impurities. TIs are promising because they possess the massless Dirac electrons which have applications in low-dissipation electronics. This lays the foundation for the development of energy efficient devices. In contrast, the suppression from bulk states may influence the topological surface state and break the linear dispersion relation between the energy and momentum. The magnetic impurities can also break the time-reversal symmetry and gapless surface state. This poses a challenge for topological surface regulation and synthesis. In the experimental work, the working mechanism of the TI-based device should also be studied. Scientists also need to study the surface state with the Dirac electron, dynamics, electrical and magnetic properties of TI. This must promote the development of TI with the effect of researchers from all over the world.

## 6. Conclusions

In summary, this paper summarizes the latest development, preparation, doping and application of TIs. Obviously, the TI, as one of the most promising optoelectronic, magnetic, semiconductor and quantum materials, has excellent optical, electrical and magnetic properties. The TI develops rapidly. Over a decade, it has developed from 2D HgTe/CdTe quantum well to 3D TI (Bi_2_Se_3_, Bi_2_Te_3_ and Sb_2_Te_3_) and TCIs (SnTe and Pb_x_Sn_1−x_Se (Te)), and then to DSMs (ZrTe_5_ and HfTe_5_) and WSMs (type-I: TaAs, TaP, NbAs and NbP; type-II: WTe_2_ and MoTe_2_). Various preparation and doping methods have improved the crystal quality of TIs. Different impurities have promoted the properties of TIs such as superconductivity. Because of the excellent nature of the TIs, they are suitable for fabricating advanced photodetectors, magnetic devices, FETs and lasers. However, the study of TIs still has challenges. The research on TIs will continue to be improved in the future.

## Figures and Tables

**Table 1:** The properties of common TIs.

Types of Properties	Items	Bi_2_Se_3_ [52]	Sb_2_Te_3_ [53]	Bi_2_Te_3_ [54]
Physical Properties	Density	7.51 g/cc	6.44 g/cc	7.73 g/cc
	a Lattice Constant	4.14 Å	4.25 Å	4.38 Å
	c Lattice Constant	28.7 Å	30.3 Å	30.45 Å
	Molecular	654.84 g/mol	626.32 g/mol	800.76 g/mol
	Formula Units/Cell (Z)	3		
Mechanical Properties	Knoop Microhardness	167 N/mm^2^	-	155 N/mm^2^
Electrical Properties	Band Gap	0.35 eV	0.30 eV	0.21 eV
	Electron Mobility	600 cm^2^/V·s	275 cm^2^/V·s	1140 cm^2^/V·s
	Hole Mobility	-	360 cm^2^/V·s	680 cm^2^/V·s
	Carrier Type (Undoped) [55]	n	p	p
Thermal Properties	Thermal Conductivity	2.40 W/m·K	1.65 W/m·K [56]	3.00 W/m·K
	Melting Point	706 °C	622 °C	585 °C
Descriptive Properties	Color	Black	Gray	Gray
	Crystal Structure	Hexagonal-Rhombohedral Tetradymite Structure-R(-3)M [57]		
	Solubility	insoluble in organic solvents and water; soluble in strong acids [58]	Insoluble in H_2_O [59]	Insoluble in H_2_O; soluble in ethanol [59]
Number of Dirac Electron		1		
Groups of Elements		The V and VI main groups		
Type of Bond		Covalent bond (main), ion bond, Van der Waals forces		
Optimum Growth Temperature		550 °C [60]	200 °C [61]	400 °C [61]

**Table 2:** Comparison of different preparation methods.

Methods	Structure	Advantage	Disadvantage	Ref.
Mechanical Exfoliation	Layer (min 1 QL) [62]	1. Simple process 2. High crystal quality 3. Low cost	1. Difficult to control accuracy 2. Poor reproducibility 3. Uneven thickness	[63,84]
Molecular-Beam Epitaxy (MBE)	Film (min 1 nm) [65]	1. Clean growth environment, low growth temperature, slow growth rate (about 1 μm/h) 2. Good crystal integrity, accurate composition and uniform thickness 3. Easy to dope	1. Expensive equipment and high maintenance costs 2. High Vacuum requirements	[64,85,86,87,88]
Chemical Vapor Deposition (CVD)	Nanowire Nanoribbon (LT) Nanoplate (HT)	1. Simple equipment 2. High flexibility 3. A large scale of TI can be prepared on complex shapes of substrates	1. Low deposition rate 2. Need chemical safetyprotection	[89,90]
Solvothermal Synthesis	Nanowire Nanorod (min 2~3 μm) [72]	1. Simple process, easy to operate 2. Low cost 3. High crystal quality 4. Easy to dope (such as S atoms)	1. Uneven solution temperature 2. Difficult to concentrate the distribution of the reaction product particle size 3. Low yield and purity	[74,75,76]
Metal-organic Chemical Vapor Deposition (MOCVD)	Semiconductor, Thin film (min 10 μm) [77]	1. Low temperature & normal pressure or low pressure (1.33~13.3 kPa) 2. High purity, less thermal defects and intrinsic defects 3. The film thickness, composition and doping amount can be precisely controlled 4. Large scale of film, high uniformity, good repeatability, industrial production	1. Expensive equipment 2. Toxic, harmful and flammable source material 3. Cannot prepare thin films of different materials at the same time	[77,79,80]

**Table 3:** Superconductivity of different impurity-doped TIs.

Element	Chemical Formula	Doping Amount (x)	Transition Temperature (K)	Methods	Ref.
In	In_x_(Pb_1−y_Sn_y_)_1−x_Te	>0.1 (y = 0.35~1.0)	4.7	TSFZ	[103]
	In_x_(Pb_0.5_Sn_0.5_)_0.7_Te	0.3	4.7	FZ	[104]
	In_x_Sn_1−x_Te	0.045	0.37	VTM	[109]
	In_x_Sn_1−x_Te	0.5	4.7	MBM	[110]
	In_x_Sn_1−x_Te	0.5	2.8 (P = 2.5 GPa)	MBM	[111]
	In_x_Sn_1−x_Te	0.45	4.5	TSFZ	[112]
	In_x_Sn_1−x_Te	0.45	3.8-BaF_2_ (100) 3.6-BaF_2_ (111)	PLD	[113]
	In_x_Sn_1−x_Te	0.4	4.1	MBM	[114]
Cu	Cu_x_Bi_2_Se_3_	0.12~0.15	3.8	MGM	[99]
	Cu_x_Bi_2_Se_3_	0.12~0.15	3.5~3.6	MGM	[98]
	Cu_x_(PbSe)_5_(Bi_2_Se_3_)_6_	1.47	2.9	MBM	[115]
Sr	Sr_x_Bi_2_Se_3_	0.05, 0.08, 0.12	~2.5	MGM	[116]
	Sr_x_Bi_2_Se_3_	-	2.8	MGM	[117]
Nb	Nb_x_Bi_2_Se_3_	0.25	1.8	MGM	[118]
Ag	Ag_x_Sn_1−x_Te	0.15~0.25 (optimal x = 0.2)	2.4	STR	[119]
	(Ag_x_Pb_1−x_Se)_5_(Bi_2_Se_3_)_3_	0.2, 0.22	1.7	STR	[96]
TI	TI_x_Bi_2_Se_3_	0.6	2.28	MGM	[120]
Pd	Pd_x_Bi_2_Te_3_	0.15, 0.3, 0.5, 1	5.5	MBM	[101]

**Table 4:** Comparison of photodetectors based on TIs and graphene.

Materials	Band (nm)	R (A·W^−1^)	t_r_/t_d_	D* (cm·Hz^1/2^·W^−^^1^)	PM	Structure	Substrate	Ref.
Bi_2_Te_3_–Si heterostructure	370–118,000	1	<100 ms/ <100 ms	2.50 × 10^11^	PLD	Film	Si	[136]
Sb_2_Te_3_	980	21.7	238.7 s/ 203.5 s	1.22 × 10^11^	MBE	Film	Sapphire	[137]
WS_2_–Bi_2_Te_3_ heterostructure	370–1550	30.7	20 ms/ 20 ms	2.30 × 10^11^	PLD	Film	Si	[138]
Bi_2_Se_3_–Si heterostructure	~300–1100	24.28	2.5 μs/ 5.5 μs	4.39 × 10^12^	PVD	Film	Si (100)	[139]
SnTe	405–3800	3.75	0.31 s/ 0.85 s	-	MBE	Film	Bi_2_Te_3_/STO	[140]
Bi_2_Se_3_ (NWs)–Si heterostructure	380–1310	~1000	45 ms/ 47 ms	-	VLS	NW	SiO_2_/Si	[141]
SnTe/Si heterostructure	300–1100	2.36	2.2 μs/ 3.8 μs	1.54 × 10^14^	PVD	Film	SiO_2_/Si	[20]
graphene/Si heterojunctions	400–900	0.435	1.2 ms/ 3 ms	1.4 × 10^8^	CVD	ML	Cu	[142]
graphene	532–10,000	8.61	~100 s/ 100 s	-	MEM	ML	SiO_2_/Si	[133]
metal-graphene-metal (MGM)	300–6000	0.0061	-	-	-	ML/BL	Si	[143]
Graphene oxide Vertical junction	290–1610	0.0236	130 ms/ 152 ms	3.31 × 10^7^	HTM	Film	Si	[144]
GMG heterostructure	473–1064	0.205	24 μs/ 46 μs	-	LBLT	LBL	SiO_2_/Si	[145]
Graphene/PbS QDs	~500–1500	10^7^	10 ms/ 100 ms	7 × 10^3^	MEM	ML/BL	SiO_2_/Si	[146]
Graphene/Bi_2_Te_3_ heterojunctions	~400–1500	35	8.7 ms/ 14.8 ms	-	CVD	NP	SiO_2_/Si	[147]

**Table 5:** Comparison of different FETs.

Materials	I_on/off_	I_DS Max_ (μA/μm)	Reference
Bi_2_Se_3_-Film	10^4^	1.1 × 10^3^	[165]
Stannanane-iodine	10^4^	10^2^	[166]
Bi_2_Se_3_-Nanowire	10^8^	10	[167]
TI-FMTJ	10^4^	-	[173]
TI-Ribbon (2D)	10^2^	10^3^	[175]
TI-Film	-	6 × 10^3^	[176]
TI-FMTJ(double)	10^4^	-	[177]
SnTe-Film	10^6^	0.6	[178]
(Bi_1__−__x_Sb_x_)_2_Se_3_-Film	250	-	[179]
WSe_2_-hBN	10^7^	10^3^	[174]
Si-Fin-PSG	-	<10^2^	[180]
Ge/Si Heterojunction	10^7^	<10^3^	[181]
Graphene-LPE	10^5^	-	[182]
Graphene-Nanoribbon	10^4^	<10^2^	[183]
Graphene-ZnO microwire	55	<10	[184]

**Table 6:** Different mode-locked fiber lasers based on TIs and some normal materials.

Materials	Pulse Duration (fs)	ΔT (%)	Repetition Rate (MHz)	I_sat_ (MW/cm^2^)	S/N (dB)	Wavelength (nm)	Ref.
Sb_2_Te_3_	125	6	22.2	31	65	1558	[189]
Sb_2_Te_3_	128	6	22.32	31	65	1565	[191]
Sb_2_Te_3_	270	6	34.58	31	70	1560	[195]
Sb_2_Te_3_	70	-	95.4	-	65	1542	[196]
Sb_2_Te_3_	170	13	25.38	-	68	1558	[197]
Sb_2_Te_3_	380	-	17.07	-	67	1039.4	[198]
Bi_2_Se_3_/PVA	-	3.4	9.75	31.5	45	1565.16 & 1565.66	[192]
			8.805		75	1566.6 & 1567.2	
			433.8		45	1562.78 & 1563.35	
Bi_2_Se_3_/PVA	-	3.8	1.086	25	62	1566	[193]
Bi_2_Se_3_/PVA	359	4.6	46.4	-	58	1557–1660	[194]
Bi_2_Se_3_/PVA	22,000	3.8	8.83	25	55	~1568	[199]
Bi_2_Se_3_/PVA	500	2.4	26	-	58	1562	[200]
Bi_2_Se_3_/PVA	660	3.9	12.5	12	55	1557.5	[201]
Bi_2_Se_3_/PVA	-	3.8	8.95	30	50	~1527–1532	[202]
Bi_2_Te_3_	600	15.7	15.11	-	-	1547	[185]
Bi_2_Te_3_	448	20.56	17.76	17.46	76	1565.9	[203]
Bi_2_Te_3_	985,000	-	11.4	-	35	1560	[204]
Bi_2_Te_3_	1300	16.3	388 239	-	-	1557.4 1559.4	[205]
Bi_2_Te_3_	1320	4.8	232–390	-	60	1564	[206]
Bi_2_Te_3_	230,000	1.8	1.44	-	77	1060	[207]
Bi_2_Te_3_	-	16.2	1.1	24.6	64	1064	[208]
Bi_2_Te_3_	320	6.2	2950	28	-	1562.4	[209]
Bi_2_Te_3_	630–700	3.75	14.07–773.85	-	46.3–63	1555.9	[210]
Bi_2_Te_3_	1210	95.3	1.21	480	-	1554–1564	[211]
Bi_2_Te_3_	2490	1.7	2040	-	-	1558.5	[212]
Bi_2_Te_3_	795	20.6	27.9	-	76	1935	[213]
Bi_2_Te_3_ (n-type) Bi_2_Te_3_ (p-type)	400/392 495/385	3.6/5.7 3.1/5.4	80	21/24 25/29	-	800 1570	[214]
Bi_2_Te_3_/PMMA	4720	10.39	10.71	6.48	72.3	1548.2–1570.1	[215]
Bi_2_Se_3_	960,000	19.1	2.5	14.9	60	1064.47	[186]
Bi_2_Se_3_	908 245	5	202.7 7.4	-	80	1554.65 1563	[187]
Bi_2_Se_3_	-	5.57	-	-	-	1529.96	[188]
Bi_2_Se_3_	824	2.3	13	-	60	1560	[216]
Bi_2_Se_3_	1570	98	1.21	490	-	1557–1565	[217]
Bi_2_Se_3_	2760	4.3	640.9	-	35	1610	[218]
Bi_2_Se_3_	46,000	5.2	44.6	580	58	1031.7	[219]
Bi_2_Se_3_	-	2.11	5.03	-	-	1531.4	[220]
Bi_2_SeTe_2_	16.4 × 10^9^	61.9	8.7	4460	-	800	[221]
MoS_2_	843,000	13.6	9.67	23.1	55	1905	[222]
MoS_2_	510	2.7	463	137	-	1556.3	[223]
MoS_2_	12,700	7	88.3	-	-	1064	[224]
WS_2_	395	7.8	19.57	189	64	1560	[225]
WS_2_	1300	10.9	34.8	3.8	72	1941	[226]
WTe_2_	273	10.95	63.3	-	62	1053	[227]
WTe_2_	8600	41.2	13.987	4.56	60	2970	[228]
Graphene	19	-	107	-	55	850	[229]
Graphene	58,800	66.5	7.29	-	48	1568.1	[230]

**Table 7:** Different Q-switched fiber lasers based on TIs and some normal materials.

Materials	ΔT (%)	I_sat_ (MW/cm^2^)	E_p max_ (nJ)	P_out max_ (mW)	Wavelength (nm)	Repetition Rate (kHz)	Pulse Duration (μs)	Ref.
Bi_2_Se_3_ (Nd: Lu_2_O_3_)	-	4300	834.2	556	1077–1081	44.3–94.7	0.72–1.81	[231]
Bi_2_Se_3_	2.5	750	-	2	1562	12.3–53.7	1.6–17.7	[236]
Bi_2_Se_3_ (EDFL)	39.8	90.2	89	16.5	1560.58–1560.33	23–47	5–13	[238]
Bi_2_Se_3_ (YDFL)			6.2	1.15	1050.4	14.9–62.5	2.1–7.56	
Bi_2_Se_3_	3.7	41	313	8.4	1980	8.4–26.8	4.18–19	[239]
Bi_2_Se_3_ (Nd:LiYF_4_)	-	-	1230	198	1313.04	36.5–161.3	0.433–0.628	[240]
Bi_2_Se_3_	41.2	101.8	16	150	1545–1565.1	4.508–12.88	13.4–36	[241]
Bi_2_Se_3_	3.8	53	17.9	1.1	1067	8.3–29.1	1.95–8.3	[242]
Bi_2_Se_3_	30	4300	58.5	32	1063	~125–547	0.666–1.33	[243]
Bi_2_Se_3_	3.8	53	200	26	604	94.2–130	0.802–1.05	[244]
Bi_2_Se_3_	5	-	4700	820	1042	73–174	1.5–5	[245]
Bi_2_Se_3_/PVA	4.3	11	23.7	22.35	1565	459–940	1.9–8	[246]
Bi_2_Te_3_	-	-	278.8	3.6	1567.1	3.312–12.74	12.74–44	[203]
Bi_2_Te_3_	14.29	0.01662	1090	161	1027.9–1040.3	53.79–147.7	0.416–1.55	[232]
Bi_2_Te_3_	5.8	-	-	1.875	1559.4	8.74–21.24	4.88–8.46	[233]
Bi_2_Te_3_ (Nd:YVO_4_)	-	1.1	600	37.5	1064	~15–70	0.097	[247]
			-	46	1342	27.5–78	0.093	
Bi_2_Te_3_	30	-	4	0.044	1568	2.6–12	9.5–50	[248]
Bi_2_Te_3_	-	-	7.5	1.35	1543.3	12.6–177.7	0.217–1.2	[249]
Bi_2_Te_3_	22	57	1525	20	1510.9–1589.1	2.15–12.8	13–50	[250]
Bi_2_Te_3_	14.7	0.0046	2440 2800	111 326	1060 & 1340	37.9–45.5 75.5–116.6	0.63–1.36 0.673–1.92	[251]
Bi_2_Te_3_	17.5	-	-	247	1064	100–151.5	2–4.75	[252]
Bi_2_Te_3_	10.8	-	12.7	0.55	1560	7.5–42.8	2.81–9.36	[253]
Bi_2_Te_3_	2.5	-	38.3	2.95	1056	35–77	1–1.3	[254]
Bi_2_Te_3_	51.3	2.12	3990	327.4	2979.9	46.2–81.96	1.37–4.83	[255]
Bi_2_Te_3_	-	-	-	139.5	1557.5	31.54–49.4	3.71–5.15	[256]
Bi_2_Te_3_	-	-	5300	210	1645	14.7–40.7	6.3–15.7	[257]
Bi_2_Te_3_/PMMA	40.5	-	9300	856	2791.2	44–92	1.3–4.3	[234]
Bi_2_Te_3_/PMMA	8.8	0.64	18,300	134	1617	2.17–11.6	7.9–2.3	[235]
MoS_2_	2.15	129.4	184.7	0.77	1560	7.76–41.45	9.92–13.53	[258]
MoS_2_	27	2450	1000	50	2030	33.6–48.1	1.76	[259]
WS_2_	29.4	1.24	370	318.5	2865.7	131.6	1.73	[260]
WS_2_	7	-	28.7	8.7	635	232.7–512.8	0.207–0.65	[261]
MoSe_2_	11.4	6	35.9	115.1	1064	995–3334	0.05–0.086	[262]
MoSe_2_	4.7	3.4	42	0.79	1924	14–21.8	5.5–16	[263]
Graphene	-	-	18,000	5200	2005	73-280	0.32–1	[264]
Graphene	-	0.926	3200	2300	1063	704	0.105	[265]
Graphene	20	-	46	12	1064	140–257	0.07–0.275	[266]
